# Challenges of next‐generation sequencing in conservation management: Insights from long‐term monitoring of corridor effects on the genetic diversity of mouse lemurs in a fragmented landscape

**DOI:** 10.1111/eva.12723

**Published:** 2018-11-13

**Authors:** B. Karina Montero, Ernest Refaly, Jean‐Baptiste Ramanamanjato, Faly Randriatafika, S. Jacques Rakotondranary, Kerstin Wilhelm, Jörg U. Ganzhorn, Simone Sommer

**Affiliations:** ^1^ Animal Ecology and Conservation Hamburg University Hamburg Germany; ^2^ Institute of Evolutionary Ecology and Conservation Genomics University of Ulm Ulm Germany; ^3^ QIT Madagascar Minerals Fort‐Dauphin Madagascar; ^4^ Faculté des Sciences Université d' Antananarivo Antananarivo Madagascar

**Keywords:** corridors, fragmentation, genetic diversity, Madagascar, major histocompatibility complex, *Microcebus ganzhorni*, primates, SSCP versus NGS

## Abstract

Long‐term genetic monitoring of populations is essential for efforts aimed at preserving genetic diversity of endangered species. Here, we employ a framework of long‐term genetic monitoring to evaluate the effects of fragmentation and the effectiveness of the establishment of corridors in restoring population connectivity and genetic diversity of mouse lemurs *Microcebus ganzhorni*. To this end, we supplement estimates of neutral genetic diversity with the assessment of adaptive genetic variability of the major histocompatibility complex (MHC). In addition, we address the challenges of long‐term genetic monitoring of functional diversity by comparing the genotyping performance and estimates of MHC variability generated by single‐stranded conformation polymorphism (SSCP)/Sanger sequencing with those obtained by high‐throughput sequencing (next‐generation sequencing [NGS], Illumina), an issue that is particularly relevant when previous work serves as a baseline for planning management strategies that aim to ensure the viability of a population. We report that SSCP greatly underestimates individual diversity and that discrepancies in estimates of MHC diversity attributable to the comparisons of traditional and NGS genotyping techniques can influence the conclusions drawn from conservation management scenarios. Evidence of migration among fragments in Mandena suggests that mouse lemurs are robust to the process of fragmentation and that the effect of corridors is masked by ongoing gene flow. Nonetheless, results based on a larger number of shared private alleles at neutral loci between fragment pairs found after the establishment of corridors in Mandena suggest that gene flow is augmented as a result of enhanced connectivity. Our data point out that despite low effective population size, *M. ganzhorni* maintains high individual heterozygosity at neutral loci and at MHC II DRB gene and that selection plays a predominant role in maintaining MHC diversity. These findings highlight the importance of long‐term genetic monitoring in order to disentangle between the processes of drift and selection maintaining adaptive genetic diversity in small populations.

## 1 INTRODUCTION

Long‐term monitoring of genetic variation and the ease with which genetic data are obtained are central if we are to increase our understanding of the evolutionary consequences of anthropogenic change and to take appropriate measures to preserve the genetic diversity and adaptive potential of small and declining populations. Indeed, the assessment of genetic variation throughout time provides the means to evaluate the effects of past and current events on the demographic and evolutionary history of wild populations (Schwartz, Luikart, & Waples, 2007). However, despite their relevance in developing conservation management plans (Allendorf, Luikart, & Aitken, 2012; Leroy et al., 2017; Reed & Frankham, 2003; Schwartz et al., 2007), longitudinal datasets are still rare (Kappeler et al., 2017).

The advent of high‐throughput sequencing technologies (next‐generation sequencing, NGS) allows cost‐effective large‐scale estimates of genetic variation with the caveat that the direct comparison of the “new” with the “old” datasets can be challenging (Sommer, Courtiol, & Mazzoni, 2013). This is particularly relevant when quantifying genetic variation among complex multigene families (e.g., immune genes of the major histocompatibility complex [MHC]) because the sensitivity of sequencing methods can influence the genotyping and associated diversity patterns (Babik, 2010; Promerová et al., 2012; Sommer et al., 2013).

Major histocompatibility complex genes along with other regions of the genome associated with immune response (e.g., toll‐like receptors) are important markers providing insight into long‐term persistence of populations because they are considered to be associated with the ability of individuals to adapt to changing environments (Bonin, Nicole, Pompanon, Miaud, & Taberlet, 2007; Holderegger, Kamm, & Gugerli, 2006; Tschirren et al., 2013). MHC molecules encode peptide‐binding sites that mediate the recognition of antigens derived from intra‐ and extracellular pathogens and present them to T cells (Klein, 1986). The selection for pathogen resistance and MHC‐dependent mate choice, in combination with other evolutionary processes such as gene duplications, recombination and mutations, is the main forces shaping the extensive variability of the MHC (Bernatchez & Landry, 2003; Edwards & Hedrick, 1998; Sommer, 2005; Spurgin & Richardson, 2010).

Here, we address the challenges of long‐term genetic monitoring within the framework of a real life management scenario: the effectiveness of the establishment of corridors in restoring the population connectivity and genetic diversity of mouse lemurs. Our study focuses on a population of a newly recognized primate species, *Microcebus ganzhorni,* restricted to the forest fragments of Mandena in south‐eastern Madagascar (Hotaling et al., 2016). The littoral forests of eastern Madagascar are considered as one of the most vulnerable areas to habitat loss (Ganzhorn, Lowry, Schatz, & Sommer, 2001) with <10% of its original vegetation now being restricted to forest fragments subject to continuous degradation (Bollen & Donati, 2006; Consiglio et al., 2006; Vincelette, Théberge, & Randrihasipara, 2007). The vestigial forest fragments that remain in Mandena result from the constant rate of deforestation that occurred in the region since the 1950s and that increased between 1995 and 1998 (Bollen & Donati, 2006). The process of habitat encroachment was alleviated through the creation of a conservation zone in 1999 (Mandena Conservation Zone) as part of the environmental programme of Quebec Iron and Titanium (QIT) and QIT Madagascar Minerals (QMM). The environmental activities of QMM included the establishment of corridors aimed at restoring the connectivity between fragments. So far, knowledge of the effectiveness of corridors is limited to mark–recapture data. Previous work determined the presence of mouse lemurs within the corridors and interpatch movement has been recorded once (Andriamandimbiarisoa et al., 2015).

In order to understand the long‐term effects of fragmentation and current management strategies in restoring connectivity in Mandena, we supplement estimates of neutral genetic diversity in *M. ganzhorni* with the assessment of long‐term patterns of adaptive genetic variability of the MHC and addressed three main objectives: first, we evaluate if the recent process of habitat loss and fragmentation result in population subdivision and influence the patterns of gene flow and effective population size of *M. ganzhorni* using neutral markers. We expect that fragmentation plays a role in limiting gene flow among forest fragments and that the continuous process of habitat loss results in low effective population size as has been demonstrated for other lemur species (e.g., *Lemur catta*, Grogan, McGinnis, Sauther, Cuozzo, & Drea, 2016; *Propithecus perrieri*, Banks, Ellis, & Antonio, and Wright, 2007).

Second, we compare the patterns of neutral and adaptive genetic variability at MHC II DRB gene in *M. ganzhorni*. Evidence demonstrating contemporary selection at MHC loci is equivocal (Bernatchez & Landry, 2003; Radwan, Biedrzycka, & Babik, 2010; Spurgin & Richardson, 2010); while low variation at MHC loci has been associated with the effects of inbreeding and drift (Babik et al., 2009; Miller & Lambert, 2004; Strand et al., 2012) selection also maintains MHC variability in small populations (Aguilar et al., 2004; Miller, Allendorf, & Daugherty, 2010; Niskanen et al., 2014; Schuster, Herde, Mazzoni, Eccard, & Sommer, 2016; van Oosterhout, Joyce, & Cummings, Blais, et al., 2006). To this end, we investigate the role of contemporary selection in maintaining adaptive genetic diversity in *M. ganzhorni*.

Our last objective relates to the challenges of long‐term genetic monitoring of functional genetic diversity. We evaluate the potential discrepancies of estimates of MHC diversity obtained by a common traditional method (single‐stranded conformation polymorphism, hereafter referred to as SSCP) and high‐throughput technologies (amplicon‐based next‐generation sequencing, hereafter referred to as NGS). Prior to the establishment of corridors, work on the MHC class II DRB gene revealed a marked deficiency of heterozygotes and evidence of inbreeding (Schad, Ganzhorn, & Sommer, 2005; Schad, Sommer, & Ganzhorn, 2004). This previous work serves as a baseline for assessing, for instance, the success of conservation management approaches that aim to ensure the viability of a population. We discuss the results derived from two scenarios for which longitudinal data are available: (a) a dataset that consists of traditional (i.e., before the establishment of corridors) and NGS genotyping results (i.e., after the establishment of corridors), and (b) a dataset consisting solely of NGS genotyping results (i.e., before and after the establishment of corridors). This approach enables us to assess the influence of deriving estimates from different genotyping techniques on the way conclusions are drawn from a conservation management strategy.

## 2 METHODS

### 2.1 Study area and sample collection

**Figure 1:**
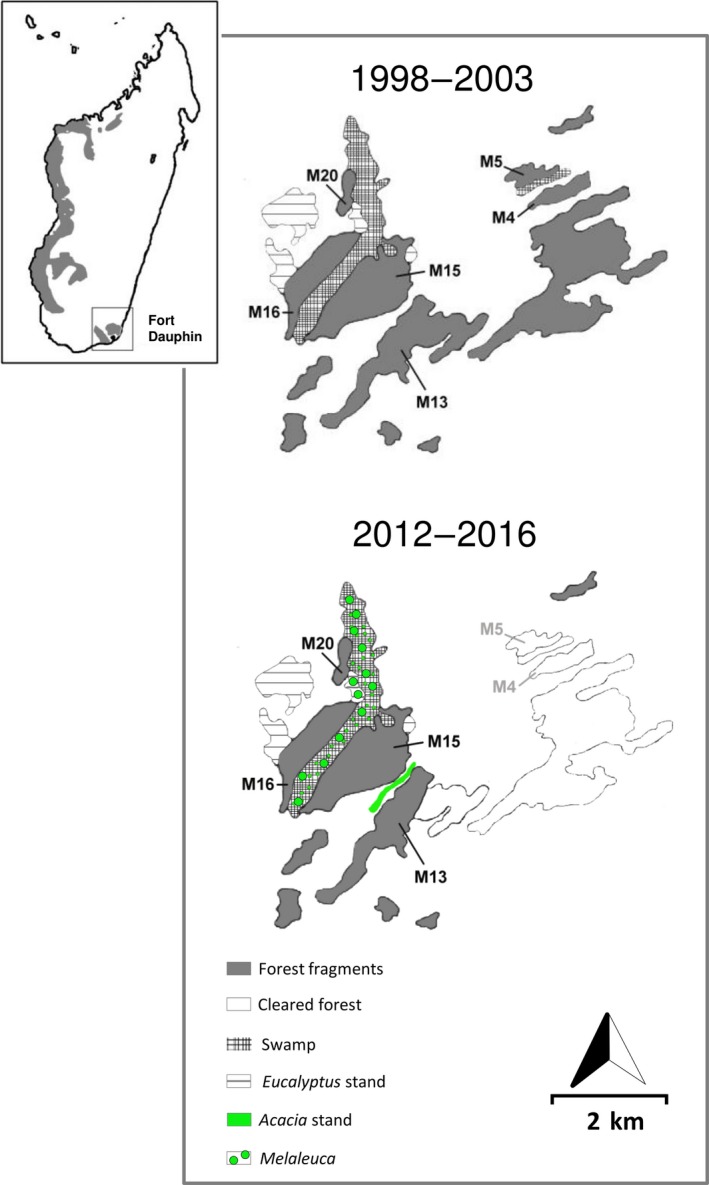
Geographical location of littoral rainforest fragments in Mandena/South‐eastern Madagascar. Sampling was conducted during two periods (before the corridor was established: 1998–2003, after the corridor was established: 2012–2016). The locations of the corridors between fragments are highlighted in green and consist of a mixture of exotic and native tree species (modified from Ramanamanjato & Ganzhorn, 2001). Forest remnants are marked in grey and are numbered. White fragments were cleared of vegetation after the first sampling period

Field work was conducted in forest fragments (labelled M4‐5, M13, M15‐16 and M20) located in the littoral rain forest of Mandena, south‐eastern Madagascar (24°56´S, 46°59´E) during two sampling periods (sampling period 1: 1998–2003, sampling period 2: 2012–2016) (Figure 1). Mouse lemurs were sampled following a standardized live‐trapping protocol explained in detail elsewhere (Ramanamanjato & Ganzhorn, 2001; Schad et al., 2004). Between 2004 and 2011, two fragments (M4 and M5) were cleared by charcoal producers. By now, the fragments M15 and M16 are legally protected as “New Protected Area.” The remaining fragments will be destroyed by mining and replaced by tree plantations and natural forest restoration. The concept and activities have been summarized in Ganzhorn et al. (2007). In 2009, as part of a restoration effort, the environmental programme of QIT and QMM established a corridor consisting of *Acacia magnum* and short (10‐ to 50‐m‐wide strip of native trees and shrubs) to connect forest fragments M13 and M15‐16. Introduced species have been chosen because they grow fast enough to produce vegetation structures that might be of use for forest species. Native trees grow too slowly. A corridor consisting of invasive *Melaleuca quinquenervia* had established itself without human intervention between M20 and M15‐16 (Andriamandimbiarisoa et al., 2015; Eppley et al., 2015). The use of corridors by mouse lemurs after 2012 has been confirmed by surveys and regular mark/recapture (Andriamandimbiarisoa et al., 2015). For analyses of genetic diversity and differentiation, we included individuals captured in forest fragments during the both sampling periods (M13, M15‐M16 and M20). The size of the forest fragments and sample sizes used for analyses are summarized in Supporting Information Table S1.

### 2.2 MHC genotyping by SSCP/Sanger sequencing

Extraction of genomic DNA from ear biopsies was carried out by using a Qiagen DNeasy Blood & Tissue Kit (Qiagen). During the first sampling period, the individual diversity of the MHC class II DRB exon 2 gene of mouse lemurs was investigated by SSCP and Sanger sequencing as detailed in Schad et al. (2004, 2005). In short, amplification was carried out by using the target‐specific primers (Schad et al., 2004) JS1 (5´‐GAGTGTCATTTCTACAACGGGACG‐3´) and JS2 (5´‐TCCCGTAGTTGTGTCTGCA‐3´). These primers flank a 171‐base pair fragment including the functionally relevant antigen‐binding sites. PCR products were denatured, loaded onto 15% polyacrylamide gels (CleanGel DNA‐HP, ETC; Elektrophoresetechnik, Kirchentellinsfurt, Germany) and run on a horizontal cooling electrophoresis system (Amersham Pharmacia Biotech, Freiburg, Germany). After DNA separation, gels were fixed and silver‐stained for DNA visualization (DNA Plus One Silver Staining Kit; Amersham Pharmacia Biotech). Samples sharing similar banding patterns were placed next to each other and re‐run on a gel. All known alleles were included on each gel and used as a reference. All identified alleles were sequenced bi‐directionally. At least three samples of each allele were excised from the gel, dissolved in 1× TBE buffer and re‐amplified under the same PCR conditions mentioned above. Cycle sequencing of the PCR products was performed by using a dye terminator sequencing kit (Applied Biosystems, Foster City, CA, USA) and then analysed by gel electrophoresis with an Applied Biosystems automated sequencer (model 377), following the manufacturer's instructions (Schad et al., 2005, 2004 ).

### 2.3 MHC genotyping by an amplicon‐based NGS approach

Samples collected during the second sampling period were also extracted by using the Qiagen DNeasy Blood & Tissue Kit (Qiagen) and the same MHC fragment was targeted. All samples from the first and second sampling periods were included in amplicon‐based NGS genotyping in two Illumina runs. The overall dataset corresponded to 684 samples genotyped from 422 individual mouse lemurs (*N*_1998–2003_: 213, *N*_2012–2016_: 209), 262 replicates and eight negative controls (Supporting Information Table S1). This allowed the comparison of the performance between traditional and NGS methods. In order to generate individually barcoded libraries, amplicons were tagged following the approach of Fluidigm System (Access Array™ System for Illumina Sequencing Systems; © Fluidigm Corporation), which required two consecutive rounds of PCR to generate amplicon libraries. In the first reaction, the 171‐base pair fragment was amplified by using the target‐specific primers described above flanked by the common sequence tags (CS1‐NNNN‐JS1) and CS2 (CS2‐JS2) required by the Fluidigm protocol. Four random base pairs were added to the forward primer to optimize cluster identification during sequencing. The second amplification reaction attached the unique sample barcodes and sequencing adapters (additional information about the laboratory procedures can be found in the Supporting Information). Paired‐end sequencing runs were performed on an Illumina MiSeq machine by using the Nanokit Reagent Kit v2 chemistry for 500 cycles. The sequencing of 692 uniquely barcoded IDs was distributed in two separate MiSeq runs.

Raw sequence data were processed by using a recently described bioinformatics work‐flow that enabled the separation of artefacts from true alleles (Santos et al., 2016; Sommer et al., 2013). The following steps were considered in the pipeline: preparation of raw files for processing, quality filtering and artefact removal and allele calling and assignment of alleles to individuals (Santos et al., 2016; Supporting Information for further details). Replicates were handled blindly throughout the genotyping pipeline. We obtained 1,794,548 reads, with a mean read coverage of 2,642 (± 1,120) reads per sample. Allele calling repeatability was 97%.

### 2.4 Microsatellites genotyping

Samples were genotyped for 14 polymorphic microsatellites: C1P3, Mm09, Mm10, Mm07 (Radespiel, Funk, Zimmermann, & Bruford, 2001), Mm22, Mm40, Mm51, Mm60, 33,103, 33,104 (Hapke, Eberle, & Zischler 2003), Mm06 (Radespiel et al., 2002), Efr56 (Jekielek & Strobeck, 1999) and C14‐2527, C20‐3430 (Buschiazzo, Beck, & Gemmell, 2011). Details of microsatellite loci and PCR laboratory conditions are summarized in Supporting Information Table S2 and in the Supporting Information, respectively.

### 2.5 Evidence of historical positive selection on MHC class II alleles

To check for the presence of codon sites exhibiting signals of historical positive selection, we used the codon‐based method (CODEML) included in the package PAML version 4.7 (Yang, 2007). Here, positive selection is indicated by a *d*_N_/*d*_S_ ratio (*ω*) > 1. The following models of codon evolution were computed: M7 (assumes a variation of *β*:* ω* among codons modelled under a *β* distribution and does not allow positive selected sites) and M8 (similar to M7 but assumes *ω* > 1). Model M7 served as a null model and was compared with model M8 by means of the likelihood‐ratio test. To identify the best fitting model, the twice log‐likelihood difference was compared with a chi‐squared distribution. Subsequently, if the model indicating selection (M8) resulted in a significantly better fit to the data, the Bayesian approach in CODEML was used to determine the identity of sites under positive selection.

### 2.6 Long‐term pattern of gene flow, genetic structure and effective population size

Genetic diversity measured by the microsatellite and MHC DRB exon 2 gene data was investigated by calculating the average number of alleles (*A*), expected (*H*_Exp_) and observed (*H*_Obs_) heterozygosity and inbreeding coefficient (*F*_IS_) by using the software package Arlequin version 3.5.2.2 (Excoffier & Lischer, 2010). Departure from Hardy–Weinberg expectations and linkage disequilibrium were checked by using GENEPOP 4.2.1 (Rousset, 2008). Genetic differentiation among fragments was assessed by means of pairwise *F*_ST_ analyses with exact tests of population differentiation based on haplotype differences. Tests were run separately for each sampling period.

Population genetic structure was evaluated by the Bayesian clustering method implemented in the program STRUCTURE version 2. 3.4 (Pritchard, Stephens, & Donnelly, 2000). To determine the likelihood of assigning individuals to *K* hypothetical populations with STRUCTURE, the model was performed on the assumption of admixture and correlated allele frequencies with no prior knowledge of sampling origin. We used the alternative ancestry prior that assumes unequal contribution of populations to the individuals sampled. To improve the performance of the MCMC sampler, we used an initial value of alpha, *α* of 0.5. These two last settings were preferred over the default parameters in STRUCTURE (uniform ancestry prior, *α* = 1) based on the recent recommendations of Wang (2017) to improve the accuracy of individual assignments to *K* populations in unbalanced datasets. The number of simulated *K* values ranged from 1 to 6, the maximum number corresponding to the number of putative populations (fragments M4‐5, M13, M16 and M20) plus 2 (Evanno, Regnaut, & Goudet, 2005), with parameters set on a Markov chain Monte Carlo of 250,000 and a burn‐in period of 100,000 for 10 replicate runs. STRUCTURE analyses were repeated across 20 subsamples to account for unequal sample sizes across fragments (Puechmaille, 2016). Following this method, we randomly subsampled individuals from the full dataset to obtain balanced input files. We subsequently run the STRUCTURE analyses using the same settings used for the full dataset and report which estimates of *K* were obtained in the majority of the analyses. Subsampling was performed by the random selection of individuals from each fragment to give the same sampling size as in the smallest sample from each period. STRUCTURE analyses were carried out by using the R package “ParallelStructure” version 1.0 (Besnier & Glover, 2013). Three different statistics were used to infer the most likely value of *K* including (a) the maximum value of the posterior probability distribution, (likelihood of *K*, ln* p*(*X*|*K*), (Pritchard et al., 2000), (b) the *ΔK* method (Evanno et al., 2005) estimated in STRUCTURE HARVESTER (Earl & vonHoldt, 2012); (c) the alternative statistics based on mean, median and maximum counts (MedMeaK, MaxMeaK, MedMedK and MaxMedK) of individuals from each fragment that form a cluster following the approach of Puechmaille (2016) and using a membership coefficient threshold of 0.5. We evaluated the correspondence between the Bayesian clustering analyses and the principal component analyses (PCA) on individual genotypes using the R package ADEGENET v.2.1.1 (Jombart, 2008).

The patterns of contemporary gene flow were examined using the program BayesAss v.1.3 (Wilson & Rannala, 2003). To estimate migration rates and the proportion of migrants (*m*) between fragments, MCMC runs were performed with 3 × 10^6^ omitting the first 1 × 10^6^ with a sampling frequency of 2000. Delta values were adjusted to optimize terminal proposed changes between chains (40%–60% of the total iterations). Convergence was verified by ensuring concordance between 10 replicate runs, each initialized with a different random seed.

We estimated effective population size (*N*_e_) for each sampling period and 95% credible limits with the linkage disequilibrium method (Waples & Do, 2010) using the program LDNE (Waples & Do, 2008). Estimates were computed for alleles that occurred with frequency values greater than *P*_Crit_ 0.02 using the parametric procedure for estimating confidence intervals. We used the temporal method (Jorde & Ryman, 1995) to estimate *N*_e_ over the entire time period (1998–2016). This method delivers reliable estimates when using datasets with more than 50 individuals and when longitudinal comparisons encompass at least five generations between samples (Waples & Yokota, 2007).

The program BOTTLENECK v. 1.2.02 (Piry, Luikart, & Cornuet, 1999) was used to examine whether populations of the littoral forest fragments of Mandena showed signals of a recent genetic bottleneck by testing for heterozygosity excess for each sampling period. Three models were employed to estimate deviations from mutation‐drift equilibrium: the stepwise mutation model (SMM), the infinite allele model (IAM) and the two‐phase model (TPM) with 90% single‐step mutations and a variance of 12. We test for a significant reduction in observed heterozygosity and the expected heterozygosity based on the number of alleles under each mutational model using the sign test of the difference (*H*_Obs_ *–H*_Exp_) and the Wilcoxon signed‐rank test with 100,000 iterations. In addition, we checked for mode shifts in the allele frequency distribution. Alleles at low frequency are likely to be lost in a population experiencing a recent bottleneck resulting in a shifted mode distribution compared with the L‐shaped distribution expected in stable populations (Cornuet & Luikart, 1996).

We examined the patterns of genetic diversity before and after the establishment of corridors by assessing the departure from mutation‐drift equilibrium (Broquet et al., 2010). For this purpose, we calculated gene diversity excess (Δ*H*) at each microsatellite locus, as the difference between observed genetic diversity (*H*_obs_) and the equilibrium gene diversity (*H*_eq_) computed under the IAM, SMM and TPM models in the software BOTTLENECK (as described above). We used linear mixed models to test whether establishing connectivity between fragments results in significant reduction of gene diversity excess (reaching equilibrium). The model was fitted with sampling period as fixed categorical effect and locus ID as a random factor using the package lme4 in R (Bates et al., 2015). Significance was assessed using type II Wald *F* tests with Kenward–Roger correction of degrees of freedom to calculate *F*‐statistics and *p*‐values. We evaluate the effect of locus and fragment on Δ*H* by calculating the adjusted repeatability (*R*_adj_, Nakagawa & Schielzeth, 2010). This was done by dividing the estimated variance component of either locus or fragment by the sum of the estimated random effect variance and estimated error variance.

### 2.7 Comparison of patterns of genetic diversity and population structure between MHC class II and neutral loci

To estimate the effect of contemporary selection at MHC, we used the Ewens–Watterson homozygosity (EW) test of neutrality (Watterson, 1978). EW compares the observed distribution of allele frequencies (*F*_obs_) to expected values of homozygosity (*F*_exp_) under the theoretical prediction that rare alleles under positive selection result in uniform frequency distributions that lie above neutral expectations. We calculated the normalized deviate of *F* (*F*_nd_) to estimate the degree to which *F*_obs_ deviate from neutral expectations. Slatkin’s (1994) method was used to test for significant deviations using 10,000 permutations in the software PyPop (Lancaster, Nelson, Meyer, Single, & Thomson, 2003). Negative values (*F*_nd_ < 0) indicate uniform frequency distributions (due to an excess of rare alleles) and suggest either balancing selection or population expansion while positive values (*F*_nd_ < 0) are interpreted as evidence of directional selection or population bottleneck (due to a deficit of rare alleles). The direct assessment of patterns of variation across MHC and microsatellite loci enables us to account for the potential role of demography.

We compared allelic diversity at MHC and microsatellite loci with respect the recent change in connectivity by estimating the mean number of distinct alleles (allelic richness, *A*_R_), the mean number of private alleles (private allelic richness, *A*_P_) and the mean number of private alleles for pairs of populations. The rarefaction method was used as implemented in the software ADZE (Szpiech, Jakobsson, & Rosenberg, 2008).

The degree to which population subdivision differed between the MHC II DRB gene and neutral microsatellite loci across sampling periods was evaluated by means of Mantel tests. We compared pairwise *F*_ST_ estimates for MHC with microsatellite *F*_ST_ whereby population differentiation is predicted to be significantly reduced for markers in which balancing selection is the main driver maintaining variability (Schierup, Vekemans, & Charlesworth, 2000). Further, we assess the relationship between pairwise *F*_ST_ estimates for MHC and geographical distance (isolation by distance, IBD) while controlling for the effect at neutral *F*_ST_ estimates (Ekblom et al., 2007). In such a test, evidence of IBD at the MHC after removing the effect of neutral differentiation rules out the influence of drift on MHC differentiation. Matrix randomizations were performed with 10,000 permutations using the package vegan package in R (Dixon, 2003). Forest fragment M4‐5 was cleared by 2012 and therefore was excluded from the analyses in which sampling periods were compared directly.

## 3 RESULTS

### 3.1 Comparison of the genotyping performance of SSCP/Sanger sequencing versus amplicon‐based NGS

The availability of samples from the first sampling period (i.e., before the establishment of corridors, Supporting Information Table S1) enabled us to evaluate the genotyping performance of traditional (*N*_SSCP_ = 227) and amplicon‐based NGS methods (*N*_NGS_ = 213). MHC II DRB genotypes for 208 individuals were generated using both methods.

SSCP/Sanger sequencing revealed the presence of 14 alleles (Mimu‐DRB*1 to Mimu‐DRB*10, Mimu‐DRB*12 to Mimu‐DRB*13, Mimu‐DRB*16, GenBank accession: AJ431266–AJ431270, AJ555835–AJ555841, AJ830740–AJ830741 Schad et al., 2005, 2004 ). Allele identities were based on 71 (41.5%) variable nucleotide positions and two insertion–deletion events at codon positions 13 and 57 (Supporting Information Figure S1).

Eleven MHC II DRB exon 2 alleles were identified by using amplicon‐based NGS. Allele identities were based on 44 (25.7%) variable nucleotide positions and one insertion–deletion event at codon position 57 (Supporting Information Figure S1). Unique amino acid sequences and the absence of stop codons suggested that all alleles encoded functional proteins. Of the 11 MHC class II alleles, seven corresponded to the alleles obtained by SSCP/Sanger analyses (Mimu‐DRB*1 to Mimu‐DRB*4, Mimu‐DRB*6, Mimu‐DRB*9 to Mimu‐DRB*10). These alleles were observed in similar frequencies to those observed by SSCP/Sanger genotyping (*F*_ST_ = 0.001, *p* = 0.352, Table 1). Two new alleles (Miga‐DRB*3 and Mimu‐DRB*38, GenBank accession: MG821085, EU137082) showed one and two base pair differences to the previously known alleles Mimu‐DRB*5 (position 147) and Mimu‐DRB*7 (positions 37 and 119) (Supporting Information Figure S1). Two additional distinct alleles were named Miga‐DRB*1 and Miga‐DRB*2 (GenBank accession: MG821083–MG821084).

**Table 1:** Major histocompatibility complex class II DRB exon 2 allele frequencies of *Microcebus ganzhorni* estimated with (A) single‐stranded conformation polymorphism (SSCP)/Sanger sequencing (*N*_Sampling period 1_: 227, GenBank accession: AJ431266–AJ431270, AJ555835–AJ555841, AJ830740–AJ830741; Schad et al., 2005, 2004 ) and (B) amplicon‐based next‐generation sequencing (NGS) (*N*_Sampling period 1_: 213, *N*_Sampling period 2_: 209; GenBank accession: MG821083–MG821085, EU137082)

	1998–2003					2012–2016				
	M4‐5	M13	M15‐16	M20	Total	M4‐5	M13	M15‐16	M20	Total
(A) SSCP										
*N*	38	29	135	25	227					
Mimu‐DRB*1	0.500	0.275	0.287	0.263	0.318					
Mimu‐DRB*2	0.130	0.225	0.133	0.211	0.153					
Mimu‐DRB*3	–	0.200	0.149	0.158	0.131					
Mimu‐DRB*4	0.019	–	0.010	–	0.009					
Mimu‐DRB*5	0.074	0.150	0.103	0.132	0.107					
Mimu‐DRB*6	0.148	0.075	0.113	0.105	0.113					
Mimu‐DRB*7	–	–	0.005	–	0.003					
Mimu‐DRB*8	–	–	0.010	–	0.006					
Mimu‐DRB*9	0.019	0.025	0.015	–	0.015					
Mimu‐DRB*10	0.074	0.050	0.062	0.105	0.067					
Mimu‐DRB*12	–	–	0.113	–	0.067					
Mimu‐DRB*13	0.019	–	–	–	0.003					
Mimu‐DRB*14	0.019	–	–	–	0.003					
Mimu‐DRB*16	–	–	–	0.026	0.003					
Private alleles	2	0	3	1	6					
(B) Amplicon‐based NGS										
*N*	37	29	122	25	213	–	67	120	22	209
Mimu‐DRB*1	0.422	0.204	0.223	0.208	0.251	–	0.306	0.217	0.275	0.252
Mimu‐DRB*2	0.109	0.167	0.112	0.146	0.123	–	0.048	0.100	0.100	0.083
Mimu‐DRB*3	–	0.148	0.107	0.167	0.103	–	0.097	0.095	0.125	0.099
Mimu‐DRB*4	0.016	–	0.013	–	0.010	–	–	0.023	–	0.013
Mimu‐DRB*6	0.125	0.056	0.094	0.083	0.093	–	0.105	0.149	0.275	0.148
Mimu‐DRB*9	0.016	0.019	0.026	–	0.020	–	0.016	0.014	–	0.013
Mimu‐DRB*10	0.063	0.093	0.086	0.083	0.083	–	0.089	0.090	0.050	0.086
Mimu‐DRB*38	–	–	0.013	0.021	0.010	–	0.032	0.032	0.025	0.031
Miga‐DRB*1	0.188	0.185	0.219	0.167	0.203	–	0.234	0.213	0.125	0.210
Miga‐DRB*2	–	–	–	0.021	0.003	–	–	0.005	–	0.003
Miga‐DRB*3	0.063	0.130	0.107	0.104	0.103	–	0.073	0.063	0.025	0.062
Private alleles	0	0	0	1	1		0	2	0	0

Four out of the five alleles (Mimu‐DRB*8, *13, *14 and *16) identified exclusively with SSCP/Sanger analysis were rare alleles and only found in a single individual each. Allele Mimu‐DRB*12, identified in 18 individuals with the SSCP/Sanger analysis, is unlikely to be an artefact but rather a contamination event. The individual carrying Mimu‐DRB*8 was no longer available for NGS analyses because of the lack of DNA.

We found agreement in individual genotypes among 49.5% of the samples. Disagreement between genotyping approaches was largely attributed to allelic dropout using the SSCP/Sanger method. Among the samples that were identified as genotyping discrepancies, 87.6% were classified as homozygous with SSCP. The dropout event of a common allele in the population (Miga‐DRB*1, Table 1) and potential false positives (Mimu‐DRB*8, *12, *13, *14 and *16) accounted for 77.2% and 20.7%, respectively, of the discrepancies in individual genotypes between the two methods.

All MHC II DRB alleles showed evidence of positive selection. Twelve and fourteen positively selected sites were identified in the allele sequences discovered by the SSCP/Sanger and the NGS approaches, respectively. The majority of the selected sites corresponded to antigen‐binding sites in humans (Brown et al., 1993; Supporting Information Table S3). Estimates of *d*_N_/*d*_S_ ratios (*ω*) were almost twofold larger among the NGS allele variants (*ω*_SSCP_ = 3.22, *ω*_NGS_ = 7.61) with more positively selected sites than those identified by the SSCP/Sanger sequencing method (Supporting Information Table S3).

Analyses of MHC DRB exon 2 genetic diversity conducted on samples from the first sampling period revealed contrasting patterns between the SSCP/Sanger sequencing and amplicon‐based NGS data. The SSCP dataset suggested marked deficiencies of heterozygotes (*H*_Obs_ = 0.38–0.54, *H*_Exp_ = 0.69–0.84, all *p* < 0.0001, Table 2) within all four fragments and strong evidence of inbreeding (*F*_IS_ = 0.38–0.54, Table 2, Schad et al., 2005, 2004 ). In contrast, data generated with amplicon‐based genotyping revealed overall higher levels of heterozygosity (*H*_Obs_ = 0.73–0.92, *H*_Exp_ = 0.73–0.87, Table 2) and showed no evidence of heterozygosity deficit. In one fragment (M15‐16), the observed heterozygosity (*H*_Obs_ = 0.91) was higher than expected (*H*_Exp_ = 0.85, *p* < 0.0001). No evidence of inbreeding was found in the amplicon‐based NGS data (*F*_IS_ = −0.07 to 0.00). Estimates of genetic diversity by using NGS data after the corridors were established (*N*_NGS_ = 262, Supporting Information Table S1) still indicated high levels of heterozygosity (*H*_Obs_ = 0.82–0.85, *H*_Exp_ = 0.81–0.85, n.s.), although slightly lower than those during the first sampling period (Table 2). No evidence of inbreeding was found (*F*_IS_ = 0.01–0.08).

**Table 2:** Genetic variation of major histocompatibility complex class II DRB exon 2 estimated by (A) SSCP/Sanger sequencing (Schad et al., 2005, 2004), (B) amplicon‐based NGS and (C) microsatellite loci

Fragment	1998–2003								2012–2016							
	*N*	*A*	*A*_R_	*A*_P_	*F*_IS_	*H*_Obs_	*H*_exp_	*p*	*N*	*A*	*A*_R_	*A*_P_	*F*_IS_	*H*_Obs_	*H*_exp_	*p*
(A) SSCP																
M4‐5	38	9	5.98	1.46	0.39	0.42	0.69	***								
M13	29	7	5.80	0.28	0.54	0.38	0.82	***								
M15‐16	135	11	6.64	1.44	0.47	0.44	0.84	***								
M20	25	7	5.79	0.56	0.38	0.52	0.83	***								
(B) Amplicon‐based NGS																
M4‐5	37	8	5.55	0.36	−0.01	0.73	0.73	n.s	–	–	–		–	–	–	–
M13	29	8	6.49	0.24	0.00	0.86	0.86	n.s	67	9	6.42	0.55	−0.05	0.85	0.81	n.s
M15‐16	122	10	6.81	0.49	−0.07	0.91	0.85	***	120	11	6.99	0.97	0.01	0.84	0.85	n.s
M20	25	9	6.97	0.85	−0.07	0.92	0.87	n.s	22	8	6.18	0.32	0.01	0.82	0.81	n.s
(C) Microsatellites																
M4‐5	40	90	4.95	0.32	0.01	0.68	0.69	n.s	–	–	–		–	–	–	–
M13	29	86	4.85	0.25	0.03	0.67	0.69	n.s	59	100	5.00	0.50	0.01	0.70	0.71	n.s
M15‐16	131	111	5.14	0.40	0.05	0.67	0.70	n.s	120	111	5.10	0.56	0.04	0.67	0.67	n.s
M20	26	83	4.69	0.34	0.04	0.65	0.68	n.s	21	82	4.81	0.48	0.08	0.63	0.69	n.s

Notes

*A*: number of alleles; *A*_P_: private alleles; *A*_R_: allelic richness; *F*_IS_: inbreeding coefficient; *H*_Obs_: observed heterozygosity; *H*_exp_: expected heterozygosity; *N*: sample size; NGS: next‐generation sequencing; SSCP: single‐stranded conformation polymorphism.

Significant deviations from Hardy–Weinberg indicated by ***p < 0.0001.

Significant population differentiation was found between fragment M4‐5 and all the other fragments sampled during the first period by both methods (*p* < 0.001, range *F*_ST SSCP_ = −0.015 to 0.54; *F*_ST NGS_ = −0.007 to 0.056, Supporting Information Table S5). Removal of the forest fragment (M4‐5) that was cleared of vegetation after the first sampling period from the analyses (Figure 1) revealed that patterns of genetic subdivision at MHC were significantly different between sampling periods (SSCP/Sanger–NGS: *F*_ST_ = 0.040, *p < *0.001; NGS–NGS: *F*_ST_ = 0.003, *p = *0.029) (Supporting Information Table S5).

### 3.2 Long‐term patterns of gene flow, bottleneck events and effective population size investigated by neutral markers

**Figure 2:**
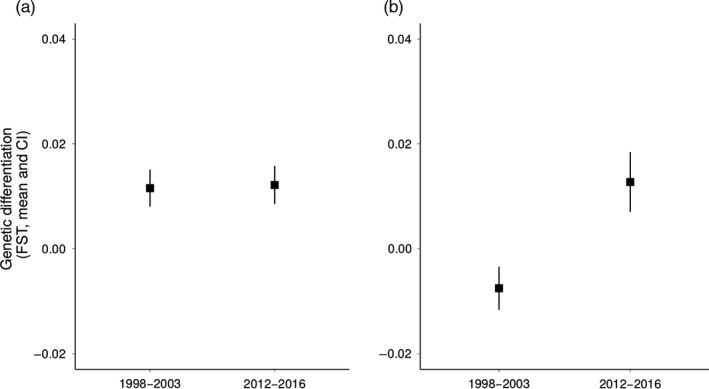
Mean (CI) genetic differentiation (*F*_ST_) for pairwise comparisons of individuals inhabiting the three forest fragments M13, M15‐16 and M20 sampled before (1998–2003) and after (2012–2016) the establishment of corridors in the littoral rain forest of Mandena. Estimates are based on (a) microsatellite loci, (b) major histocompatibility complex (MHC) DRB exon 2

The effects of fragmentation and corridors on gene flow, bottleneck events and effective population size were evaluated by monitoring the patterns of genetic change via 14 microsatellite markers. Details of microsatellite diversity estimates are provided in Supporting Information Table S4. The degree of genetic differentiation between fragments estimated by *F*_ST_ ranged from 0.010 to 0.028 before the corridors were available and from 0.005 to 0.016 after the corridors were established (Supporting Information Table S5). No evidence of populations substructure was found using the global tests of genetic differentiation among fragments (*p* = 1.00, Supporting Information Table S5). Genetic distances between fragments during the first sampling period were on average no different than those during the second period (*F*_ST_ = 0.005, *p* = 0.146). Similar results were found when removing the forest fragment (M4‐5) as this fragment has been destroyed after the first sampling period. Inclusion would skew the comparisons between the first and second period (Figure 2).

**Figure 3:**
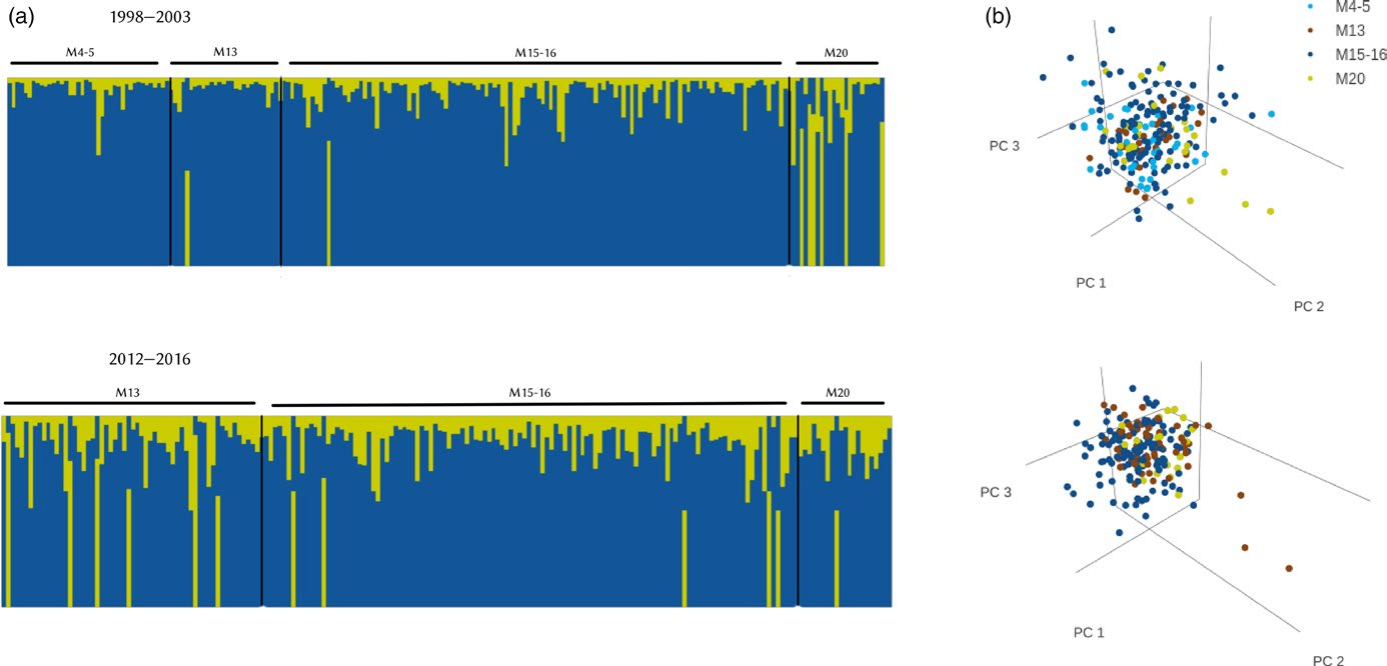
Genetic structure of *Micocebus ganzhorni* in forest fragments sampled before (top panel, 1998–2003: M4–5, M13, M15‐16 and M20) and after (bottom panel, 2012–2016: M13, M15‐16 and M20) the establishment of corridors in the littoral rain forest of Mandena. (a) STRUCTURE diagrams correspond to the full dataset with uneven sample sizes among fragments illustrating membership for each individual for *K* = 2. Each vertical line represents an individual and colours represent the inferred genetic ancestry. (b) Principal component analyses (PCA) of the full dataset created on the basis of individual genotypes of *M. ganzhorni*

Population differentiation was further investigated with the program STRUCTURE. Different criteria were used to infer the most likely number of populations for each period. Estimates of the number of ancestral clusters *K* for the full dataset (which comprised a higher number of samples but suffered from unbalanced sampling across the populations) revealed similar results across sampling periods, with a minimum of *K* = 1 (using the maximum MedMeaK and MedMedK) and a maximum of *K* = 3 (using *K* = 3, ln *p*(*X*|*K* and *ΔK* methods) ancestral clusters. With *K* = 2, we found populations of the forest fragments in Mandena consisted of mixed ancestry with no clear grouping associated with the fragment of origin (Figure 3). A pattern of admixture with no clear grouping based on location remained when setting *K* = 3 (Supporting Information Figure S2). The same patterns of membership assignment to *K* clusters were found when using the balanced datasets that had been generated through random sampling (data not shown). Population structure inferred from the randomized subsampled balanced datasets was fairly consistent across the 20 replicated analyses (Supporting Information Table S6). The majority of analyses with the 20 balanced subsampled datasets suggested *K = *1 (MedMeaK and MedMedK) or *K* = 2 (ln *p*(*X*|*K*) and *ΔK*)ancestral clusters (Supporting Information Table S6). PCA for the full dataset revealed no clear differentiation among the forest fragments (Figure 3). All together, these results favour a single population of admixed ancestry in Mandena.

Evidence of contemporary gene flow (*m*, fraction of individuals identified as immigrants) was found between some but not all fragments (Table 3). Estimates of migration rates from fragment M15‐16 to all other fragments were relatively high (*m*_1998–2006_: 0.22–0.30; *m*_2012‐2016_: 0.30). All other pairwise comparisons indicate low levels of gene flow (*m*_1998–2006_: 0.005–0.06; *m*_2012‐2016_: 0.002–0.016). Overall, these results suggest asymmetric gene flow between fragments.

**Table 3:** Mean posterior distribution of contemporary gene flow of *Micocebus ganzhorni* across forest fragments in Mandena estimated using BAYESASS

From/to	M4‐5	M13	M15‐16	M20
(A) 1998–2003				
M4‐5	**0.676**	0.013	0.035	0.012
M13	0.007	**0.678**	0.005	0.010
M15‐16	0.302	0.288	**0.898**	0.227
M20	0.013	0.020	0.062	**0.751**
(B) 2012–2016				
M4‐5	–	–	–	–
M13	–	**0.693**	0.004	0.016
M15‐16	–	0.301	**0.994**	0.303
M20	–	0.006	0.002	**0.682**

Note

Individuals belonging to a population of origin are listed in the rows. Standard deviations for all distributions were <0.05. The proportion of individuals remaining in the population of origin is listed along the diagonal (bold values).

Effective population size, calculated using the linkage disequilibrium method (Waples and Do), was similar across sampling periods, *N*_e 1998–2003_: 186 (95% CI = 154–230); *N*_e 2012–2016_: 247 (95% CI = 196–326). The *N*_e_ estimated for the overall dataset using the temporal method was 65 individuals (95% CI = 47–86).

Evidence of a bottleneck was found under the IAM model for both sampling periods, as indicated by significance in the sign and Wilcoxon tests (Supporting Information Table S7). However, tests of heterozygosity excess under the SMM and TPM models and the mode‐shift test of allelic distribution suggested that the populations in Mandena were stable. We found no significant differences in gene diversity excess (Δ*H*) before and after the establishment of corridors (Supporting Information Figure S3) independent of the model used (IAM: *F* = −0.004, *p* = 0.764; SMM: *F* = 0.006, *p* = 0.714; TPM: *F* = −0.004, *p* = 0.763). Among the random effects, the variance explained by locus was large (*R*_adj_ = 0.66–0.67), whereas the variance explained by fragment was small (*R*_adj_ = 0.003–0.03).

### 3.3 Comparison of patterns of genetic diversity and population structure between MHC class II and neutral loci

Results from the Ewens–Watterson test provide evidence of contemporary balancing selection at MHC and provide support for the role of demographic fluctuations occurring in Mandena. We found significant even distribution of allele frequencies at MHC, albeit fragment M4‐5 (1998–2003) and M20 (2012–2016) showed no significant deviations from neutrality (Supporting Information Table S8). Negative values of Ewens–Watterson statistics along with significant deviation from expected equilibrium in multiple microsatellite loci (~40%) indicate that *M. ganzhorni* exhibits a signature of population expansion in Mandena.

**Figure 4:**
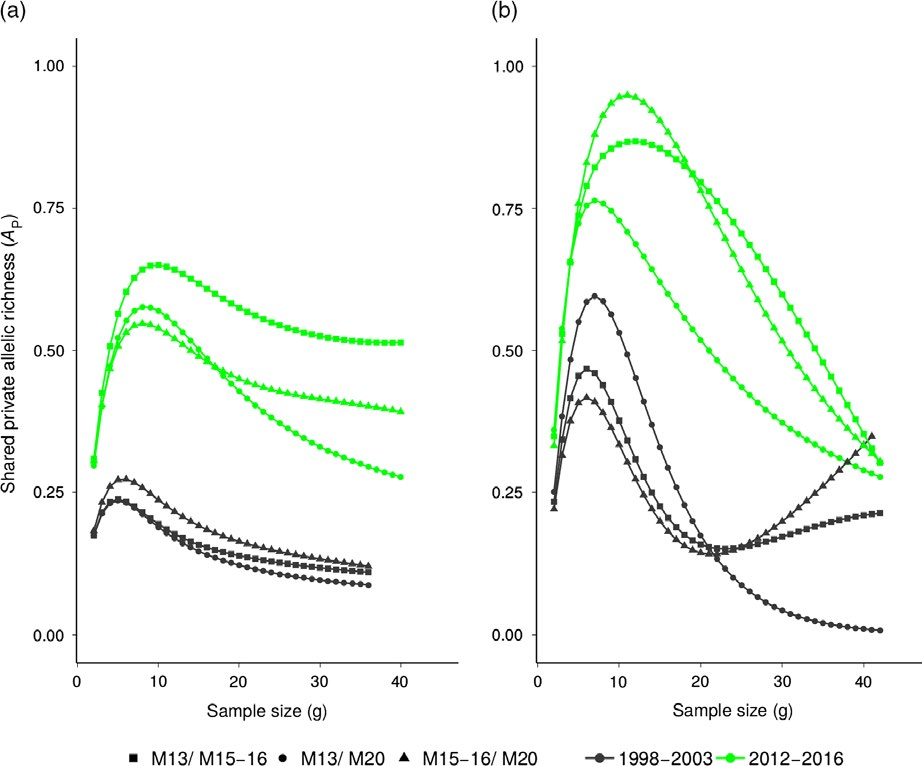
Inference of uniquely shared alleles in *Micocebus ganzhorni* between a combination of populations from forest fragments in Mandena using the rarefaction method implemented in the ADZE software (Szpiech et al., 2008). (a) microsatellite loci, (b) major histocompatibility complex (MHC) II DRB gene. Forest fragment M4‐5 was cleared by 2012 and therefore was excluded from the analyses

Allelic richness at microsatellite and MHC markers was similar among fragments and between sampling periods (Table 2, Supporting Information Figure S4). In contrast, the mean number of distinct alleles varied considerably among fragments and sampling periods at both types of markers. We found an increase in private allelic richness at forest fragments M13 and M15‐16 after the establishment of corridors. However, M20 showed no change in private allelic richness at microsatellite loci and a decrease at MHC after the establishment of corridors (Table 2, Supporting Information Figure S4). Results from analyses in which fragment pairs were combined show that overall, more shared private alleles at microsatellite loci were found for the second sampling period compared to the first sampling period (Figure 4). This suggests that forest fragments after the establishment of corridors share more their contemporary genetic history and provides evidence of ongoing gene flow. In contrast, the inference of shared private alleles at MHC is strongly affected by sample size (Figure 4). This is likely due to the low number of rare alleles at MHC and the associated susceptibility to random sampling. Despite the limitation of the effect of sample size at MHC, there is some evidence of recent gene flow between M13 and M20 (Figure 4).

We found no significant correlation between *F*_ST_ pairwise estimates at MHC and microsatellite loci for either sampling period (Mantel test_1998–2003_: *r*_M_ = 0.20, *p* = 0.45; Mantel test_2012–2016_: *r*_M_ = 1.00, *p* = 0.167). The overall estimate of population differentiation varied between sampling periods (Figure 2). For the first sampling period, we found a lower estimate at the MHC gene (*F*_ST_ = −0.007, CI = −0.012, −0.003) than at microsatellite loci (*F*_ST_ = 0.016, CI = −0.012, −0.003) whereas for the second sampling period no significant difference was found (MHC: *F*_ST_ = 0.013, CI = 0.007, 0.019; microsatellites: *F*_ST_ = 0.012, CI = 0.009, 0.016). Furthermore, no evidence of isolation by distance at MHC while controlling for neutral differentiation was found for either sampling period (partial Mantel test_1998–2003_: *r*_M_ = 1.00, *p* = 0.50; partial Mantel test_2012–2016_: *r*_M_ = 1.00, *p* = 0.50), suggesting that drift is not a major driver of MHC differentiation among forest fragments.

## 4 DISCUSSION

In this study, we aimed at understanding the effects of habitat loss and fragmentation on the genetic diversity of *M. ganzhorni,* a species of mouse lemurs that, to date, is considered to be restricted to the remnant littoral forests of south‐eastern Madagascar (Hotaling et al., 2016). The extensive process of habitat degradation documented since the 1950s along with estimates of low adaptive genetic diversity (Schad et al., 2005, 2004 ) led to the a priori expectation that mouse lemurs in Mandena were vulnerable to the detrimental effects of drift and inbreeding. With this in mind, corridors were established to provide quick recovery of the habitat within the protected area of Mandena and to restore connectivity between forest fragments considered to be isolated from each other. Contrary to our initial hypothesis, we find that fragmentation did not limit the occurrence of gene flow within Mandena. Despite evidence of low effective population size, our results suggest that selection is a main force maintaining adaptive variability at MHCII DRB gene. We discuss how long‐term genetic monitoring of *M. ganzhorni* enables the role that process of fragmentation plays on the population dynamics and evolutionary history of Mandena and consider these processes when evaluating the value of corridors as a conservation strategy. We address our findings by first commenting on the challenges associated with comparing “old” (i.e., SSCP/Sanger sequencing) with “new” (i.e., NGS) datasets delivered by the rapid development of sequencing technologies and their implications for the assessment of management strategies. We then discuss the evolutionary history of the population of *M. ganzhorni* in Mandena as suggested by genetic differentiation, the patterns of gene flow and effective population size. Finally, we evaluate the long‐term effects of fragmentation on genetic diversity at MHC DRB II gene and the influence of the establishment of corridors on neutral and adaptive genetic variability.

### 4.1 Genotyping performance of traditional SSCP/Sanger compared with amplicon‐based NGS

One major purpose of our study was to evaluate to what degree genotyping results of the MHC II DRB gene assessed with traditional methods (SSCP/Sanger sequencing) were comparable with the data generated with high‐throughput technologies (amplicon‐based NGS, Illumina). This issue is relevant in the context of the long‐term monitoring of small populations for which previous studies involving traditional tools act as a baseline for future investigations that aim to inform management decisions. In our study, the individual genetic diversity (*H*_e_) was greatly underestimated (by about 50%) by using the traditional SSCP/Sanger sequencing method.

Our results revealed that allelic dropout had important effects on estimates of population genetic diversity. The common allele (Miga‐DRB*1, 20.3%) identified by amplicon‐based Illumina sequencing was not resolved by SSCP. The latter largely explains why *H*_Obs_ estimated by SSCP was strikingly lower than *H*_Obs_ estimated by Illumina, since 35% of the heterozygotes with Miga‐DRB*1 were misassigned as homozygotes by SSCP. At the population level, however, more MHC II DRB alleles were detected by SSCP (14 alleles) than by the amplicon‐based NGS (11 alleles). The latter is in contrast to previous work in which a lower number of alleles were detected by SSCP compared with NGS (Promerová et al., 2012; Sommer et al., 2013).

Despite the lower allelic diversity revealed by NGS (and associated smaller power of analysis, *N*_SSCP_ = 14, *N*_NGS_ = 11), estimates of d_N_/d_S_ ratios (*ω*) were almost twofold larger among the NGS allele variants (*ω*_SSCP_ = 3.22, *ω*_NGS_ = 7.61) with more positively selected sites being identified by the latter method (Supporting Information Table S3). These results suggest that the role of historical balancing selection maintaining the variation of the MHC II DRB gene of *M. ganzhorni* is even stronger than would be estimated with traditional genotyping methods. Details of the methodological aspects influencing the discordance in genotyping between traditional and NGS are discussed in detail in the Supporting Information.

Given the lack of agreement between genotyping methods, an important question remains: to what degree does the inclusion of the “old” with the “new” data influence the interpretation of our findings? Considering a scenario in which the effect of corridors is evaluated by using a dataset consisting of SSCP/Sanger results before corridors (Schad et al., 2005, 2004 ) and amplicon‐based NGS for the period after which corridors were established, we find an increased *H*_Obs_ and no signal of inbreeding after the establishment of corridors. From these results, connectivity can be argued to have enabled the restoration of adaptive genetic diversity. In contrast, a dataset with amplicon‐based NGS genotyping results for both sampling periods suggests that adaptive diversity remained unchanged, despite the efforts of establishing connectivity between forest fragments. However, agreement is found in both sets of data with respect to the patterns of genetic differentiation in our longitudinal comparison. Fragment M4‐5 is most distinct from the other fragments (Supporting Information Table S5) and, when comparing estimates of genetic differentiation of the three fragments for which data were available from both sampling periods, we find contrasting patterns in genetic differentiation between forests fragments after the establishment of corridors (these results are further discussed below; Supporting Information Table S5).

The identification of discrepancies when different methodological approaches are used is not surprising; on the contrary, it is an expected outcome given the distinct sensitivity of a particular approach. Standard methods to meet reproducibility are flagships in science. Our findings highlight that longitudinal studies of complex gene families, such as the MHC, require the genotyping of “old” together with “new” samples by the current NGS sequencing platform in order to attain improved estimates of adaptive variability.

### 4.2 Long‐term patterns of gene flow, bottleneck events and effective population size investigated by neutral markers

A major outcome of this study is that fragmentation has a limited effect on dispersal of mouse lemurs among the fragments of Mandena. We find asymmetrical patterns of migration, with evidence that Mandena conforms a “core‐satellite” scenario in which the largest forest fragment, M15‐16, appears to act as a source population of dispersing individuals to the smaller neighbouring fragments. This pattern of gene flow was observed during both sampling periods implying that dispersal by mouse lemurs between forest fragments took place prior to the establishment of corridors, a result that remained elusive from our capture–mark–recapture data (Andriamandimbiarisoa et al., 2015; Blanthorn, 2013).

High gene flow is also congruent with the absence of population structure and with our findings of similar levels of heterozygosity and allelic richness (Table 2, Supporting Information Figure S4) over the 18‐year time span considered in this study. Estimates of population genetic structure indicate that *M. ganzhorni* in Mandena corresponds to a single population of admixed ancestry (Figure 3). This pattern of genetic ancestry can be interpreted as arising either as a signature of shared ancestry previous to the onset of population divergence (Pamilo & Nei, 1988) or as a recent event of admixture between multiple founder populations (Muir & Schlötterer, 2005; Sampson et al., 2015) or as being an indication that the population is located at the edge of a spatial expansion (Excoffier & Ray, 2008; Rius & Darling, 2014). The determination of which evolutionary trajectory better explains the patterns of admixed ancestry requires extensive temporal and spatial sampling (Li et al., 2008; Sarno et al., 2017; Yoder et al., 2016) and is outside the scope of our study.

Considering a single population in Mandena, effective population size was estimated to be low: the temporal method indicates an *N*_e_ of about 65 individuals and results for each sampling period indicate that *N*_e_ remained constant over time. Low effective population size can result from frequent population turnover or from drift (Østergaard, Hansen, Loeschcke, & Nielsen, 2003). The remarkable congruence between the sampling periods indicates that low estimates of effective population size result from drift rather than frequent population turnover. Whether what appears to be a reduction of effective population size in *M. ganzhorni* is linked to the process of habitat modification induced by human activities or historic events remains to be explored, particularly in light of the influence of climate and sea‐levels oscillations during the Holocene shaping the littoral forests of south‐eastern Madagascar (Virah‐Sawmy, Willis, & Gillson, 2009).

Long‐term effects of fragmentation in mouse lemurs in Mandena were detected through the signatures of a population bottleneck under the IAM model occurring during both sampling periods, though under the SMM and TPM models, considered to simulate more closely microsatellite mutation (Di Rienzo et al., 1994; Estoup & Cornuet, 2000; Piry et al., 1999), no evidence of a recent bottleneck was detected. Evidence of population stability given the restricted current distribution of *M. ganzhorni* and the historical habitat reduction in the region should be interpreted with caution. First, the low effective population size of *M. ganzhorni* found in Mandena does not imply a demographic collapse. Demography in species with a polygynous mating system is only affected to a minor degree mainly as a consequence of variance in breeding success and sex ratios. Second, immigration can cancel out gene diversity excess (Δ*H*) (Keller et al., 2001; McEachern, Vuren, Floyd, May, & Eadie, 2011) and hamper the signal of a genetic bottleneck. We demonstrate that migration occurs among forest fragments, providing evidence of the dispersal potential of mouse lemurs in a fragmented landscape and suggest that the population of Mandena most likely illustrates a scenario of partial rather than complete isolation. Lastly, we find that genetic diversity excess (Δ*H*) remained unchanged during the 18‐year time span covered in our study (Supporting Information Figure S3). In their simulations, Broquet et al. (2010) attribute a constant behaviour in Δ*H* to the loss of both genetic diversity and equilibrium gene diversity in a similar rate, particularly in a setting of high immigration. In line with this observation, the patterns of gene diversity excess found in *M. ganzhorni* provide further evidence that Mandena was and perhaps remains part of a larger meta‐population.

### 4.3 Effect of fragmentation and establishment of corridors on long‐term patterns of neutral and adaptive genetic diversity

A central goal in our study was to find out if and how the establishment of corridors as a management strategy is effective in alleviating or reversing the pressures that fragmentation and habitat loss exert on mouse lemurs in Mandena. As mentioned earlier, the implementation of corridors in Mandena was based on the assumption that fragmentation represented a dispersal barrier to mouse lemurs as suggested from mark–recapture data. In light of our post hoc finding that genetic exchange took place despite fragmentation, do we detect any effects on neutral and adaptive genetic diversity after the establishment of corridors?

Our comparison between the two sampling periods spanning close to 18 years of genetic monitoring demonstrates the high levels of genetic diversity in terms of heterozygosity in both the MHC II DRB gene and microsatellite markers before and after the establishment of corridors (*H*_Exp_, Table 2). Evidence of low genetic diversity derived from neutral markers with respect to a reduction in fragment size has been found in the Milne‐Edwards' sportive lemur, *Lepilemur edwardsi* (Craul et al., 2009), in the golden‐brown mouse lemur, *Microcebus ravelobensis* (Guschanski, Olivieri, Funk, & Radespiel, 2007) and postulated for Tattersall's sifaka (*Propithecus tattersalli*) (Quemere, Amelot, Pierson, Crouau‐Roy, & Chikhi, 2012). Apart from these three examples, neutral genetic diversity appears to be maintained despite discontinuities of species within their geographical range, for example mouse lemurs *Microcebus bongolavensis*,* Microcebus danfossi* (Olivieri, Sousa, Chikhi, & Radespiel, 2008) and *Microcebus murinus* (Fredsted, Pertoldi, Schierup, & Kappeler, 2005; Radespiel, Sarikaya, Zimmermann, & Bruford, 2001; Wimmer & Kappeler, 2002) and redfronted lemurs (Wimmer & Kappeler, 2002). Rapid population turnover as a result of high mortality rates (Goodman, O'Connor, & Langrand, 1993), relatively large litter size (Lahann, Schmid, & Ganzhorn, 2006) and a highly promiscuous mating system (Andrès & Solignac, 2003; Eberle & Kappeler, 2006) might prevent inbreeding and therefore buffer the effects of small population size.

Concerning the role of contemporary selection on MHC II DRB gene, results from the Ewens–Watterson test evaluating within population diversity provide evidence that the demographic history of mouse lemurs in Mandena is characterized by recent population expansions as indicated by multiple microsatellite loci deviating significantly from neutral expectations (Supporting Information Table S8). In a context of population expansion, we find that contemporary balancing selection maintains variability at MHC II DRB gene within fragments M13 and M15‐16. The absence of a signature of balancing selection in some fragments (M4‐5 and M20 during the second sampling period) suggests that these sites might be more susceptible to demographic history (Meyer, Single, Mack, Erlich, & Thomson, 2006).

We compared measures of population differentiation (*F*_ST_) between MHC II DRB gene and microsatellites to tease apart the role of selection and drift maintaining adaptive diversity in *M. ganzhorni*. The absence of a correlation between pairwise *F*_ST_ at MHC and microsatellite markers and the lack of evidence of isolation by distance when controlling for neutral differentiation confirm that selection, rather than drift, is a main driver of MHC variability. Intriguingly, the pattern of MHC differentiation varied between sampling periods. Before the establishment of corridors, MHC differentiation falls well below that found at microsatellite loci. This is an expected outcome for adaptive loci because balancing selection acts as a homogenizing force (Hedrick, 1998; Muirhead, 2001; van Oosterhout, 2009), yet few studies provide evidence consistent with this prediction (Mona et al. 2008; van Oosterhout Joyce, & Cummings, 2006; van Oosterhout, Joyce, & Cummings, Blais, et al., 2006. Novel alleles brought in by immigration are likely to result in a common pool of MHC genes among forest fragments in Mandena as has been suggested for guppy populations in Trinidad (van Oosterhout Joyce, & Cummings, 2006; van Oosterhout, Joyce, & Cummings, Blais, et al., 2006).

Results after the establishment of corridors show a contrasting pattern whereby similar levels of MHC and microsatellite differentiation suggest that either balancing selection is weak or unequal selective pressures among forest fragments slow down the rate in which MHC alleles are exchanged (Muirhead, 2001). Distinguishing between these two scenarios in a context of restored connectivity requires further research. Other factors, such as reproductive strategies (i.e., MHC‐disassortative mate choice) and parasite‐driven selective pressures are likely to play important roles in maintaining diversity in *M. ganzhorni*, as has been shown in *M. murinus* (Huchard, Baniel, Schliehe‐Diecks, & Kappeler, 2013; Schwensow, Eberle, & Sommer, 2008). Our long‐term monitoring of MHC II DRB revealed some changes in the allele frequencies of specific alleles across the 18 years. Future research will determine whether and in what way the long‐term patterns of functional diversity of the MHC gene have been shaped by pathogen pressures.

Insight into the role of corridors influencing the patterns of genetic diversity is apparent by examining the evolution of private alleles at microsatellite loci. We find that although we show that gene flow among fragments occurred during the first sampling period, more shared private alleles at neutral markers were found among forest fragments after the establishment of corridors in Mandena (Figure 4). The contrasting patterns of shared private alleles at neutral markers between sampling periods provide evidence that secondary forest consisting of exotic plants enhances the movement of mouse lemurs and augments gene flow among forest fragments. Furthermore, it seems that some corridors are more effective, as suggested by the larger number of shared private alleles between forest fragments M13 and M15‐16 compared to the other fragment pairs.

Contrary to the microsatellite data, detecting a signature of gene flow through the assessment of the evolution of private alleles at MHC II DRB gene is not straightforward. Fewer rare alleles at the population level are maintained at MHC so larger sample sizes become necessary to overcome the constraints of random sampling. The low number of rare alleles found in *M. ganzhorni* is likely to be associated with the role of drift after the event of a severe population reduction at some point during the evolutionary history of the species (Sutton, Nakagawa, Robertson, & Jamieson, 2011). Despite the limitation of sample size, we find more shared private alleles between fragments M13 and M20 during the second sampling period (Figure 4). Yet, gene flow is unlikely to result from the establishment of corridors because these two fragments lay further apart (Figure 1). The results are probably influenced by the translocation of 14 individuals from M13 to M20 by people in 2014 (J.B. Ramanajato and E. Refaly, unpublished data). These individuals were not included in our analyses so the translocation cannot be reconstructed but, if the animals had been able to establish themselves in their new environment, this would have reduced the differences between populations. Although we did not directly test if genetic diversity can be traced back to the translocation, our results suggest that diversity might be improved remarkably fast (<2 years) at MHC II DRB gene. Although the value of translocations as a conservation tool has focused in the demographic effects (Frankham, 2015; Ujvari & Belov, 2011), immunogenetic rescue appears to be a promising tool to restore MHC diversity (Grogan, Sauther, Cuozzo, & Drea, 2017; Grueber et al., 2017; Madsen, Shine, Olsson, & Wittzell, 1999).

Until a recent species reassessment (Hotaling et al., 2016), mouse lemurs found in Mandena were recognized as a disjunct population of the grey mouse lemur *M. murinus* (Weisrock et al., 2010). The widespread distribution of *M. murinus* together with its flexible feeding behaviour and habitat use (Mittermeier, Hawkins, & Louis, 2010) has led to the assumption that the species is more robust to disturbance than its congeners (Dammhahn & Kappeler, 2008). *M. ganzhorni* shows little evidence of reduced adaptive genetic diversity at the individual level with estimates of heterozygosity comparable with those found in larger populations of *M. murinus* (Huchard et al., 2012; Schwensow, Dausmann, Eberle, Fietz, & Sommer, 2010). At the population level, however, the variability of MHC II DRB in terms of the number of alleles detected in *M. ganzhorni* in Mandena is significantly lower compared with the variability of MHC II DRB of larger populations of *M. murinus*. Huchard et al. (2012) found 61 alleles by using an NGS approach (454 pyrosequencing, *N* = 665), whereas we recovered 11 alleles (Illumina, *N* = 422). Low MHC allelic diversity is probably associated with the much smaller population size of mouse lemurs in Mandena (Babik, Durka, & Radwan, 2005; Bollmer, Vargas, & Parker, 2007; Gangoso et al., 2012) as the study population in Kirindy (assessed by Huchard et al., 2012) resides within a bloc of several thousand hectares whereas Mandena comprises a few hundred hectares at the most. Despite our findings of low effective population size of *M. ganzhorni* in Mandena, we provide evidence of the role of historical and contemporary selection maintaining diversity at MHC II DRB gene. These findings are in agreement with the comprehensive assessment of the effects of population reductions on MHC variability in that while it appears that polymorphism is severely compromised in small populations (Eimes et al., 2011; Sutton et al., 2011) as is the case for mouse lemurs in Mandena, selection plays an important role in maintaining diversity at the MHC II DRB gene (Radwan et al., 2010).

## 5 CONCLUSIONS

Outcomes of our study echo previous work that emphasize the relevance of long‐term genetic monitoring that capture both neutral and adaptive variability. In doing so, it is possible to disentangle the processes of demography and selection maintaining adaptive genetic diversity. We also point out that in light of the rapid turnover in sequencing technologies, results delivered by less sensitive methods can lead to erroneous conclusions when comparing results from traditional methods with results delivered by new generation sequencing.

Long‐term monitoring enabled us to assess the value of corridors as a conservation strategy, a tool that has long been debated (Simberloff, Farr, Cox, & Mehlman, 1992; Tewksbury et al., 2002). Our results confirm mouse lemurs in Mandena appear to be robust to the effects of fragmentation and that the effect of corridors is masked by the dispersal abilities of this species. Notwithstanding, we find through the assessment of the evolution of shared alleles at neutral markers that after the establishment of corridors gene flow was increased.

All together, we show that the interplay between immigration and gene flow among forest fragments has shaped the patterns of genetic variability of mouse lemurs in Mandena. The processes reconstructed in the present paper must have been confined mainly within the littoral forest fragments between Mandena and Lokaro, some 20 km northeast of Mandena. The fragments there are separated by heath‐type vegetation and spaced a few hundred to a few kilometres (Ganzhorn et al., 2007). The evolution of these forest fragments is still unclear (Virah‐Sawmy et al., 2009). There used to be a direct link to the closest bloc of natural forest towards the west at least until some 40 years ago (Martin, 1972), but the populations of *Microcebus* found in evergreen rain forest west of the littoral forest belongs to a different species (*M. tanosi*; Hotaling et al., 2016). The exchange along the coast towards the west must have been hampered for many generations as it is blocked by the town of Fort Dauphin.

Despite evidence of low effective population size, *M. ganzhorni* maintains high levels of individual genetic diversity. Although unexpected, similar results have been reported in a variety of lemur species and interpreted as a signature of past population dynamics (e.g., *Varecia variegata,* Baden et al., 2014; *P. perrieri*, Salmona et al., 2015; *Indri indri,* Nunziata et al., 2016). A scenario where small populations harbour moderate to high genetic diversity is promising for timely conservation efforts. At least some of the extant lemur species could thus exchange individuals and maintain genetic diversity in an anthropogenic landscape composed of natural forest fragments and agroforestry (Gérard, Ganzhorn, Kull, & Carrière, 2015; Irwin et al., 2010; Steffens, Rakotondranary, Ratovonamana, & Ganzhorn, 2017).

## CONFLICT OF INTEREST

None declared.

## Data Availability

Data available from the Dryad Digital Repository: https://doi.org/10.5061/dryad.2fh81pd.
