# Supplementary material for: Challenges of next‐generation sequencing in conservation management: Insights from long‐term monitoring of corridor effects on the genetic diversity of mouse lemurs in a fragmented landscape
Source: Evol Appl. 2018 Nov 13;12(3):425–42. doi: 10.1111/eva.12723 (PMC6383737; doi:10.1111/eva.12723)
Supplement: Supplementary file 1 [file EVA-12-425-s001.pdf]

Challenges of NGS in conservation management: insights from long-term monitoring of corridor effects on the genetic diversity of mouse lemurs in a fragmented landscape

B. Karina Montero[1,2], Ernest Refaly[3], Jean-Baptiste Ramanamanjato[3], Faly Randriatafika[3], S. Jacques Rakotondranary[4], Kerstin Wilhelm[2], Jörg U. Ganzhorn[1], Simone Sommer[2]

Contents

1. Supplementary laboratory procedures

1.1 MHC genotyping by SSCP/Sanger sequencing

1.2 Amplicon-based NGS

1.3 Microsatellite genotyping

2. Bioinformatic workflow for amplicon-based NGS data

3. Methodological aspects influencing genotyping performance of traditional SSCP/Sanger compared with amplicon-based NGS

4. Supplementary data

4.1 Table S1. Summary of sample sizes.

4.2 Table S2. Details of microsatellite loci and PCR multiplex conditions.

4.3 Table S3. Tests of historical positive selection.

4.4 Table S4. Microsatellite diversity estimates

4.5 Table S5. Estimates of population differentiation of *M. ganzhorni.*

4.6 Table S6. Estimate of the number of STRUCTURE *K* clusters.

4.7 Table S7. Results from BOTTLENECK for the populations

4.8 Table S8. Ewens-Watterson results

4.9 Figure S1. Alignment of MHC class II alleles detected in *M. ganzhorni*

4.10 Figure S2. Genetic structure of *M. ganzhorni.*

4.11Figure S3. Gene diversity excess (ΔH) versus predicted equilibrium gene diversity (H_{eq})

4.12 Figure S4. Allelic richness and private allelic richness

5. Supplementary references

1. Supplementary Laboratory Procedures

MHC genotyping by SSCP/Sanger sequencing: amplification of the MHC class II DRB exon 2 was carried on with target-specific primers (Schad, Sommer, & Ganzhorn, 2004) JS1: 5´ -GAGTGTCATTTCTACAACGGGACG-3´ and JS2: 5´-TCCCGTAGTTGTGTCTGCA-3´). PCR products were denatured, loaded onto 15% polyacrylamide gels (CleanGel DNA-HP, ETC. Elektrophoresetechnik, Kirchintellinsfurt, Germany) and run on a horizontal cooling electrophoresis system (Amersham Pharmacia Biotech, Freiburg, Germany). After DNA separation, gels were fixed and silver-stained for DNA visualization (DNA Plus One Silver Staining Kit, Amersham Pharmacia Biotech, Freiburg, Germany). Samples sharing similar banding patterns were placed next to each other and re-run on a gel. All known alleles were included on each gel and used as a reference. All identified alleles were sequenced bi-directionally. At least three samples of each allele were excised from the gel, dissolved in 1xTBE buffer and re-amplified under the same PCR conditions mentioned above. Cycle sequencing of the PCR products was performed by using a dye terminator sequencing kit (Applied Biosystems, Foster City, CA, USA) and then analysed by gel electrophoresis with an Applied Biosystems automated sequencer (model 377), following the manufacturer´s instructions (Schad, Ganzhorn, & Sommer, 2005; Schad et al., 2004).

Amplicon-based NGS library preparation: amplicon libraries were generated using a two-step PCR approach. The first PCR round was carried out in a 10-µl reaction volume, including 1 µl DNA template, 0.5 µM primers, 1 µl GC enhancer and 1 unit AmpliTaq Gold 360 Master Mix. PCR cycling included an activation step at 95°C for 10 min followed by 30 cycles consisting of a denaturation step at 95 °C for 30 s, annealing at 57 °C for 30 s and elongation at 72 °C for 60 s. A final elongation step was omitted to reduce artefact formation (Smyth et al., 2010). The second PCR contained 2 µl of the product generated by the initial PCR, 1 µl GC enhancer, 80 nM per barcode primer and 0.5 units AmpliTaq Gold 360 Master Mix in a final volume of 20 µl. Cycling conditions were the same as those outlined above but the number of cycles was reduced to six. PCR products

were purified by using an Agilent AMPure XP (Beckman Coulter) bead cleanup. The size and concentration of the cleaned PCR products were estimated with the QIAxcel Advanced System (Qiagen). Cleaned samples were pooled at equimolar ratios into a single tube. The amplicon library was prepared for sequencing according to the MiSeq Reagent Kit Preparation Guide (Illumina, San Diego, CA, USA) and spiked with a 5% Phix library.

Microsatellite genotyping: the microsatellite loci were fluorescently labelled by using the M13 method (Schuelke, 2000) and amplified under optimized multiplex PCR combinations based on allele size distribution. PCR was performed in a total volume of 7.5 µl containing 0.56 µl primer mix, 0.13 µM M13 primer labelled with a fluorescent tag (either FAM,YAKYE, ATTO-565 or ATTO-550), 0.75 µl DNA template, 0.75 µl Q solution and 1 U Qiagen Multiplex PCR Master Mix. A step-down cycling profile was employed under the following conditions: 95 ºC 15 min, 12 X (94 ºC 30 sec, locus-specific initial annealing temperature 90 sec, 72 ºC 60 sec), 24 X (94 ºC 30 sec, locus-specific final annealing temperature 90 sec, 72 ºC 60 sec), 1 X 60 ºC 30 min. Products were sized on an ABI 3130 Genetic Analyzer (Applied Biosystems) and genotypes were scored via GeneMapper version 4.0 (Applied Biosystems). Micro-checker version 2.2.3 (Van Oosterhout, Hutchinson, Wills, & Shipley, 2004) was used to test for the presence of null alleles, scoring errors and allele dropout.

2. Bioinformatic Workflow for Amplicon-Based NGS Data

Raw sequence data was processed following the pipeline fully described in Santos et al., (2016). Paired-end reads were merged with FLASH ((Magoč & Salzberg, 2011)) by using optimized minimum and maximum overlap parameters (-m= 150, -M= 230) and an offset phred-score of 30. Adapters and consecutive stretches of Ns were trimmed by using a customized Python script. Here, only reads with a perfect match to the forward and reverse target primers (JS1 and JS2) were kept for further analyses. Clear artefacts (repeats of A, C, T, G, GC or GT motifs longer than 15 bp) and reads that had more than 90% of their bases below a 30 phred quality score were removed by using the software FASTX-Toolkit. Chimaera detection and removal were performed by using the default settings of UCHIME (Edgar et al., 2011). Reads were then submitted to a local BLAST against a database built with publicly available MHC class II sequences fetched from GenBank. A dedicated python script was used to parse the BLAST results and to filter out reads that fell below an e-value threshold of 10^{-13}. This e-value minimizes the inclusion of sequences that don't correspond to the MHC (e.g. Phix) in the following steps. Individual fasta files were pooled into a single file and an alignment of all reads was used for allele calling by using Oligotyping (Eren et al., 2015). This approach allowed the discrimination of similar alleles based on the identification of subtle single nucleotide variants resulting from a Shannon entropy analysis. Oligotyping (v. 2.2) was performed by using those positions (components) with higher-than-background entropy suggesting polymorphic sites. These components were identified based on visual inspection of the distribution of entropy in the alignment. Alleles were subsequently mapped to individuals by using a customized Python script (Santos et al., 2016).

3. Methodological aspects influencing genotyping performance of traditional SSCP/Sanger compared with amplicon-based NGS

Our comparison between a traditional method (SSCP/ Sanger) with amplicon based NGS identified type I and type II errors (i.e. allelic dropout as well as false positives). Additional alleles detected with SSCP can be attributed to the shortcomings of stabilizing the denatured DNA and the establishment of optimal conditions that allow maximum gel resolution (Glavač & Dean, 1993; Hayashi & Yandell, 1993; Ortí, Hare, & Avise, 1997). Variations in the run temperature, the PCR volume loaded onto the polyacrylamide gel and the amount of glycerol added to the gel mix are some of the factors that influence the banding pattern and might result in extra or faint bands (Sunnucks et al., 2000). The absence of sequences of these rare alleles among the putative variants obtained by high coverage NGS suggests that they are probably PCR-based artefacts such as chimeric products (Brakenhoff, Schoenmakers, & Lubsen, 1991; Meyerhans, Vartanian, & Wain-Hobson, 1990; Smyth et al., 2010) and Taq DNA polymerase errors (Qiu et al., 2001).

Alelic dropout, defined here as any mechanism that underestimates heterozygosity or the presence of an allele, was the main driver of the discrepancies in the individual genotypes reported in our study. Allelic dropout can be caused by PCR competition which can often be resolved by high sequencing depth using NGS. However, an additional mechanism of allelic dropout that can occur using methods like SSCP is band sharing (i.e. very similar conformation of an allele that results in overlapping bands). Suboptimal resolution in the banding pattern of SSCP gels might also explain underestimates of individual heterozygosity.

The ability to generate more sequencing data per individual (i.e. high sequencing depth) represents a clear advantage of NGS over traditional methods employed for genotyping MHC genes, although the processing of larger datasets is associated with numerous challenges (Babik, 2010; Lighten, van Oosterhout, & Bentzen, 2014). Limitations of NGS include platform-dependent sequencing errors; for instance, Illumina reports up to 0.1% error rates for 75-85% of bases (Glenn, 2011) and, as in SSCP/Sanger sequencing, PCR biases are a pervasive concern in NGS analysis (Burri, Promerová, Goebel, & Fumagalli, 2014; Lenz & Becker, 2008). The need to distinguish true

allelic variation from artefacts has pushed forward the development of many bioinformatic pipelines that aim at increasing the accuracy of MHC genotyping (e.g. Huchard et al., 2012; Lighten et al., 2014; Radwan et al., 2012; Sebastian, Herdegen, Migalska, & Radwan, 2016; Sommer, Courtiol, & Mazzoni, 2013). Our estimates of MHC II allelic diversity in *M. ganzhorni* by using the amplicon-based NGS approach offer increased confidence over traditional methods because of high-sequencing depth (2642 ± 1120 reads per individual) relative to the number of MHC II DRB loci (Galan et al., 2010), repeatability (97%) and a stringent bioinformatic workflow (Santos et al., 2016; Sommer et al., 2013).

4. Supplementary data

Table S1. Summary of sample sizes used for microsatellites and MHC class II DRB exon 2 genotyping by SSCP / Sanger sequencing and amplicon-based NGS.

Fragment	Sampling period	Size (ha)	*N*	N msats	N SSCP	N NGS	Nb. of PCR replicates	Total reads	Mean read coverage	
									Raw	Cleaned
M4-5	1998-2003	69	40	40	38	37	27	227558	2050 (±1034)	1857 (±912)
M13	1998-2003	109	29	28	29	29	12	233972	3079 (±1883)	2582 (±1061)
	2012-2016	68	65	57	--	65	35	530353	2867 (±876)	2539 (±771)
M15-16	1998-2003	148	136	125	135	123	89	878933	2170 (±866)	1988 (±803)
	2012-2016	148	122	122	--	122	60	1030581	3086 (±992)	2739 (±894)
M20	1998-2003	6	26	24	25	25	19	222548	2588 (±1039)	2296 (±896)
	2012-2016	6	22	21	--	22	20	238686	3141 (±1216)	2810 (±1109)
Total	1998-2016	332	440	417	227	423	262	3362631	2642 (±1120)	2359 (±942)

N, number of individuals sampled.

Nb. of PCR replicates, number of independent PCR replicates used to assess genotyping repeatability of amplicon-based NGS.

Mean (± SD) read coverage: raw, sequences passing filtering criteria according to standard procedures of the Illumina platform; cleaned, sequences kept for allele calling with Entropy and Oligotyping analysis.

Table S2. Details of microsatellite loci and PCR multiplex conditions. Locus identity, fluorescent dye, initial and final annealing temperatures for step-down cycling conditions, forward-M13 and reverse primer concentrations in the PCR, allele size ranges and number of alleles

Locus	Dye	Initial T_a (C°)	Final T_a (C°)	[F-M13] (μM)	[R] (μM)	Size range	*N* alleles
C14-2527	YAKYE	54	62	0.05	0.09	237-264	9
C20-3430	ATTO550	54	62	0.05	0.09	171-216	7
Mm07	ATTO550	65	57	0.02	0.04	270-296	8
Mm09	FAM	65	57	0.04	0.08	182-232	11
Mm40	ATTO550	66	58	0.02	0.05	159-190	6
Mm43b	ATTO565	64	56	0.03	0.06	168-172	5
33103	YAKYE	55	47	0.04	0.08	86-94	4
33104	YAKYE	55	47	0.03	0.06	284-324	8
C1P3	YAKYE	55	47	0.03	0.06	220-270	10
Efr56	ATTO565	55	47	0.03	0.06	254-278	3
Mm06	ATTO550	55	47	0.02	0.04	160-208	11
Mm10	FAM	55	47	0.03	0.06	136-178	10
Mm22	ATTO565	55	47	0.02	0.03	226-250	5
Mm51	ATTO550	55	47	0.02	0.03	114-134	3
Mm60	FAM	55	47	0.03	0.06	108-152	8

Table S3. Log-likelihood values and parameter estimates of models testing for selection processes acting on MHC DRB exon 2 of *Microcebus ganzhorni* revealed by SSCP / Sanger sequencing and amplicon-based NGS.

Model	lnL		Estimates of parameters		Positively selected sites	
	SSCP	NGS	SSCP	NGS	SSCP	NGS
Model M7	-1024.46	-721.69	p=0.009<br>q=0.063	p=0.008<br>q=0.058	Not allowed	Not allowed
Model M8	-1006.43	-695.56	p_0=0.965<br>(p_1=0.034)<br>p=0.088<br>q=0.921<br>ω=3.215	p_0=0.967<br>(p_1=0.033)<br>p=0.011<br>q=0.061<br>ω=7.613	**5F****,**7E****, 13R*, **16F****, **17V****, **26Y***, 36D**, **40W****, 46I**, **50E****, **53K***, **57Y****	**5F****, **7E****, **9Y****, 13R**, **16F****, **17V****, **26Y****,36D**, **40W****,46I**, **49D****, **50E****, **53K****, **57Y****

Parameters p and q computed from the beta distribution. $\omega = d_N/d_S$ ratio. p_n = proportion of sites that fall into ω_n site class.

Site positions inferred to be under positive selection estimated at a * 95% and ** 99% confidence interval level. In bold are sites known as antigen binding for MHC class IIB exon 2 in humans (Brown *et al.* 1993)

Table S4. Number of alleles and heterozygosity estimates of 15 microsatellite loci in *M. ganzhorni*. The locus Mm43b was excluded from subsequent analyses because of evidence for the presence of null alleles. Individual genotypes with missing data on more than 4 microsatellite loci (2%) or with low amplification success (4%) were excluded from the overall dataset. A total of 417 samples yielded genotype data at 11-14 microsatellite loci.

		M4-5			M13			M15-16			M20		
Locus	N alleles	*N*	Ho	He	*N*	Ho	He	*N*	Ho	He	*N*	Ho	He
33103	4	4	0.63	0.60	4	0.76	0.69	4	0.69	0.67	4	0.72	0.68
33104	11	6	0.70	0.73	9	0.74	0.77	11	0.80	0.76	6	0.68	0.69
C14-2527	10	6	0.74	0.74	9	0.89	0.84	9	0.79	0.82	8	0.75	0.86
C1P3	12	9	0.84	0.82	10	0.80	0.79	12	0.80	0.83	9	0.83	0.84
C20	10	5	0.69	0.76	7	0.76	0.74	9	0.65***	0.72	6	0.70	0.79
Efr56	3	3	0.50	0.57	3	0.44	0.55	3	0.51	0.53	3	0.38	0.47
Mm06	12	9	0.90	0.77	11	0.80	0.77	12	0.81	0.79	12	0.72	0.73
Mm07	8	8	0.63	0.79	7	0.47	0.80	8	0.41***	0.80	7	0.36***	0.80
Mm09	11	11	0.88	0.85	10	0.87	0.87	11	0.83	0.83	9	0.80	0.83
Mm10	14	9	0.88	0.82	10	0.84	0.86	14	0.85	0.85	11	0.85	0.85
Mm22	5	5	0.73	0.71	5	0.72	0.66	5	0.61	0.61	5	0.55	0.54
Mm40	6	5	0.65	0.69	6	0.74	0.72	6	0.69	0.72	6	0.87	0.78
Mm43b	5	6†	0.51*	0.72	7†	0.52***	0.78	6†	0.48***	0.77	6†	0.26***	0.73
Mm51	3	2	0.05	0.05	3	0.07	0.07	3	0.14	0.14	3	0.13	0.17
Mm60	11	8	0.74	0.77	9	0.75	0.72	11	0.77	0.77	7	0.66	0.62

Significant deviations from Hardy-Weinberg indicated by *P<0.01, ***P<0.0001, † Null alleles

Table S5. Estimates of population differentiation of *M. ganzhorni* caught before and after the corridors were established in littoral forest fragments of Mandena. Estimates from A) SSCP / Sanger sequencing and B) amplicon-based NGS.

A) SSCP

	1998-2003			
	M4-5	M13	M15-16	M20
M4-5		0.008	0.009	0.006
M13	0.053*		0.413	0.920
M15-16	0.054***	0.007		0.356
M20	0.053*	-0.015	0.008*	

B) Amplicon-based NGS

	1998-2003				2012-2016			
	M4-5	M13	M15-16	M20	M4-5	M13	M15-16	M20
M4-5		0.002	0.045	0.032	-	-	-	-
M13	0.056***		0.432	0.572	-		0.666	0.359
M15-16	0.032***	-0.003		0.908	-	0.003		0.736
M20	0.038***	-0.014	-0.007		-	0.019*	0.011	

C) Microsatellites

	1998-2003				2012-2016			
	M4-5	M13	M15-16	M20	M4-5	M13	M15-16	M20
M4-5		1.000	1.000	1.000	-	-	-	-
M13	0.009*		1.000	1.000	-		0.186	0.659
M15-16	0.010***	0.007*		1.000	-	0.005***		0.572
M20	0.028***	0.016*	0.009*		-	0.015***	0.016*	

Pairwise F_{ST} are given below the diagonal nad the *P*-values of exact tests for population differentiation above the diagonal. Levels of significance (F_{ST}) are denoted by *P<0.01, ***P<0.0001,

Table S6. Population structure of Microcebus ganzhorni in Mandena during two sampling periods. Estimate of number of STRUCTURE K clusters are inferred from multiple statistics: i) supervised estimates based on the count of independent clusters (median of means,MedMeaK, maximum of means MaxMeak, median of medians, MedMedK, maximum of medians, MaxMedK, (Puechmaille, 2016); ii) natural logarithm of the probability of the data (Ln Pr(X|K), Pritchard, Stephens, & Donnelly, 2000); and iii) ΔK ((Evanno, Regnaut, & Goudet, 2005)).

	1998-2003						2012-2016					
	MedMeaK	MaxMeak	MedMedK	MaxMedK	Ln Pr(X|K)	ΔK	MedMeaK	MaxMeak	MedMedK	MaxMedK	Ln Pr(X|K)	ΔK
Balanced datasets												
K=1	20	20	20	18	2	-	20	18	20	19	19	_
K=2	0	0	0	2	17	20	0	2	0	1	3	11
K=3	0	0	0	0	1	0	0	0	0	0	0	7
K=4	0	0	0	0	0	0	0	0	0	0	0	2
K=5	0	0	0	0	0	0	0	0	0	0	0	0
K=6	0	0	0	0	0	0	0	0	0	0	0	0
Full dataset												
K=	1	2	1	1	3	3	1	1	1	1	1	3

Table S7. Results from BOTTLENECK for the populations of *M. ganzhorni* in Mandena sampled during two sampling periods. P-values are shown for sign and Wilcoxon tests (one-tailed) of heterozygosity excess under three mutation models together with the distribution shape of alleles inferred from the mode-shift test.

	1998-2003			2012-2016		
	SMM	IAM	TPM	SMM	IAM	TPM
Wilcoxon test	0.941	<0.001	0.452	0.923	<0.001	0.643
Sign test	0.070	0.004	0.446	0.069	0.004	0.552
Mode-shift test	L-shape			L-shape		

Table S8. Ewens-Watterson results for a) before and b) after the establishment of corridors in Mandena . Slatikin's exact tests to compute F_{exp} , F_{obs} and p-values were computed using 10000 replicates.

a) 1998-2003

Locus	M4-5					M13					M15-16					M20				
	A	F_{exp}	F_{obs}	F_{nd}	*P*	A	F_{exp}	F_{obs}	F_{nd}	*P*	A	F_{exp}	F_{obs}	F_{nd}	*P*	A	F_{exp}	F_{obs}	F_{nd}	*P*
33103	4	0.55	0.40	-0.91	0.22	4	0.53	0.36	-1.05	0.07	4	0.62	0.38	-1.28	0.09	4	0.52	0.40	-0.77	0.09
33104	6	0.41	0.28	-0.95	0.08	8	0.29	0.24	-0.52	0.35	9	0.35	0.23	-0.91	0.27	6	0.36	0.28	-0.70	0.51
C14-2527	6	0.41	0.27	**-1.03**	**0.04**	7	0.34	0.19	**-1.32**	**0.04**	9	0.35	0.17	**-1.44**	**0.04**	8	0.28	0.16	**-1.39**	**0.02**
C1P3	9	0.28	0.19	-0.99	0.13	9	0.26	0.21	-0.61	0.26	10	0.33	0.18	**-1.19**	**0.01**	7	0.32	0.19	**-1.31**	**0.01**
C20	6	0.41	0.24	**-1.29**	**0.02**	5	0.45	0.25	**-1.40**	**0.01**	9	0.35	0.22	-1.03	0.20	7	0.32	0.23	-0.87	0.34
Efr56	3	0.66	0.44	-1.23	0.07	3	0.64	0.45	-1.09	0.13	3	0.72	0.44	**-1.47**	**0.05**	3	0.63	0.50	-0.74	0.16
Mm06	9	0.29	0.24	-0.51	0.17	7	0.33	0.25	-0.76	0.06	11	0.30	0.20	-0.87	0.11	8	0.28	0.32	0.43	0.56
Mm07	8	0.32	0.22	-0.90	0.23	6	0.38	0.22	**-1.33**	**0.02**	8	0.39	0.18	**-1.46**	**0.04**	6	0.37	0.21	-1.34	0.06
Mm09	11	0.23	0.16	-0.90	0.10	10	0.23	0.13	-1.36	0.07	10	0.33	0.18	**-1.22**	**0.02**	9	0.25	0.17	-0.99	0.06
Mm10	9	0.29	0.19	-1.01	0.06	7	0.34	0.22	-1.06	0.11	13	0.26	0.15	-1.10	0.09	7	0.32	0.19	**-1.25**	**0.02**
Mm22	5	0.48	0.29	**-1.18**	**0.05**	5	0.45	0.35	-0.70	0.25	5	0.55	0.41	-0.75	0.07	3	0.62	0.61	-0.03	0.35
Mm40	5	0.48	0.32	**-1.04**	**0.03**	6	0.38	0.36	-0.19	0.35	6	0.48	0.28	**-1.18**	**0.04**	6	0.35	0.22	**-1.25**	**0.02**
Mm51	2	0.80	0.95	0.90	0.69	2	0.78	0.96	1.10	0.77	3	0.71	0.91	1.01	0.75	3	0.62	0.83	1.25	0.83
Mm60	8	0.32	0.24	-0.71	0.14	7	0.34	0.37	0.29	0.63	11	0.30	0.23	-0.61	0.14	7	0.32	0.32	-0.08	0.51
DRB_{NGS}	8	0.31	0.28	-0.28	0.35	8	0.29	0.16	**-1.45**	**0.01**	10	0.32	0.15	**-1.41**	**0.01**	9	0.25	0.15	**-1.33**	**0.04**

b) 2012-2016

Locus	M13					M15-16					M20				
	A	F_{exp}	F_{obs}	F_{nd}	*P*	A	F_{exp}	F_{obs}	F_{nd}	*P*	A	F_{exp}	F_{obs}	F_{nd}	*P*
33103	4	0.58	0.30	**-1.54**	**0.02**	4	0.61	0.29	**-1.74**	**0.01**	4	0.58	0.30	**-1.52**	**0.01**
33104	8	0.34	0.23	-0.87	0.41	11	0.29	0.24	-0.46	0.52	4	0.34	0.23	-0.71	0.18
C14-2527	9	0.31	0.15	**-1.40**	**0.02**	9	0.35	0.20	**-1.12**	**0.03**	8	0.31	0.15	-1.08	0.07
C1P3	10	0.28	0.23	-0.52	0.35	12	0.27	0.17	-0.93	0.21	8	0.28	0.23	**-1.11**	**0.01**
C20	7	0.38	0.27	-0.83	0.13	9	0.35	0.35	-0.01	0.69	5	0.38	0.27	**-1.49**	**0.01**
Efr56	3	0.68	0.45	-1.21	0.08	3	0.71	0.51	-1.06	0.09	3	0.68	0.45	-0.10	0.62
Mm06	11	0.25	0.23	-0.25	0.75	12	0.27	0.22	-0.50	0.14	10	0.25	0.23	0.22	0.57
Mm07	7	0.38	0.21	**-1.26**	**0.01**	7	0.43	0.23	-1.29	0.05	7	0.38	0.21	-0.63	0.09
Mm09	9	0.31	0.15	**-1.41**	**<0.01**	10	0.32	0.16	**-1.29**	**0.03**	6	0.31	0.15	**-1.39**	**0.01**
Mm10	10	0.28	0.13	**-1.47**	**0.02**	11	0.29	0.15	**-1.33**	**0.02**	11	0.28	0.13	-0.42	0.34
Mm22	5	0.50	0.34	-0.94	0.12	5	0.54	0.37	-0.93	0.06	5	0.50	0.34	-0.74	0.21
Mm40	6	0.43	0.27	-1.11	0.10	6	0.48	0.29	**-1.14**	**0.01**	5	0.43	0.27	**-1.29**	**0.03**
Mm51	3	0.67	0.93	1.38	0.86	3	0.71	0.80	0.49	0.38	2	0.67	0.93	0.33	0.49
Mm60	8	0.34	0.25	-0.71	0.25	10	0.32	0.24	-0.66	0.34	4	0.34	0.25	0.00	0.25
DRB	9	0.31	0.19	**-1.05**	**0.02**	11	0.29	0.15	**-1.32**	**0.02**	8	0.31	0.19	-0.67	0.30

A, number of alleles. Fexp , expected homozygozity Fobs., observed homozygozity. Fnd , normalized deviate. P , p-value one tailed test (adjusted for multiple comparisons using the BY method, Benjamini & Yekutieli 2001)

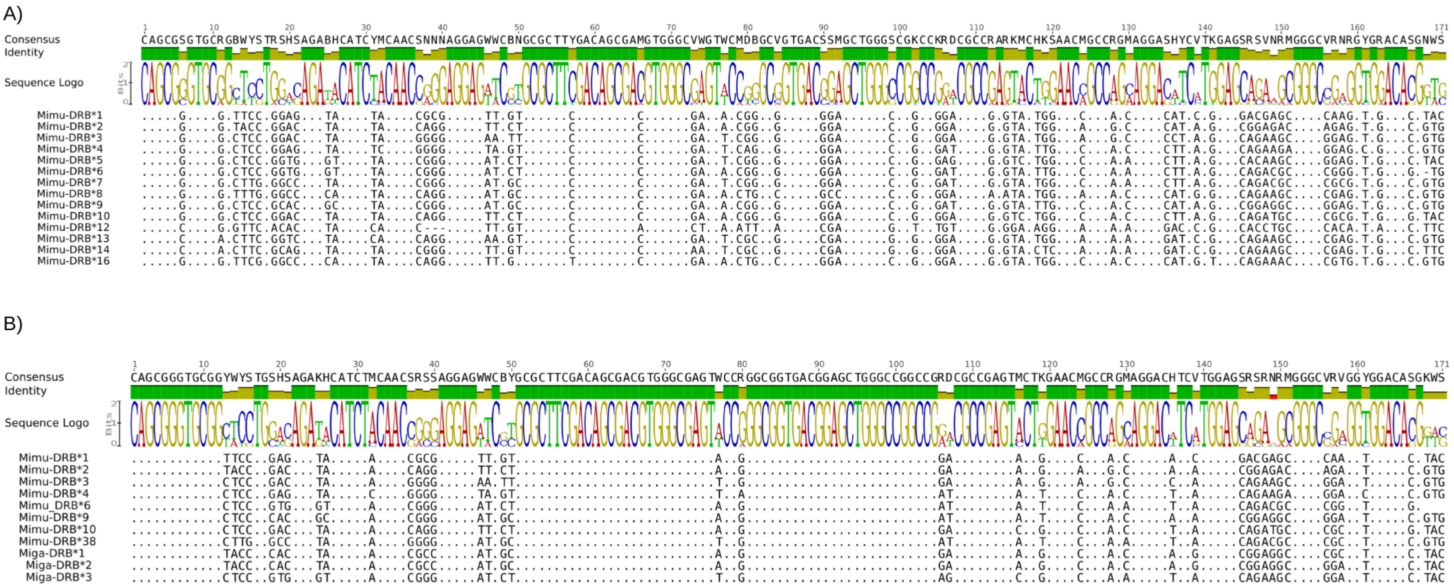

Figure S1. Alignment of MHC class II alleles detected in *M. ganzhorni* with A) SSCP / Sanger sequencing ($N_{1998\text{-}2003}$: 227, GeneBank accession: AJ431266-AJ431270, AJ555835–AJ555841, AJ830740–AJ830741) and B) amplicon-based NGS ($N_{1998\text{-}2003}$: 213, $N_{2012\text{-}2016}$: 209).

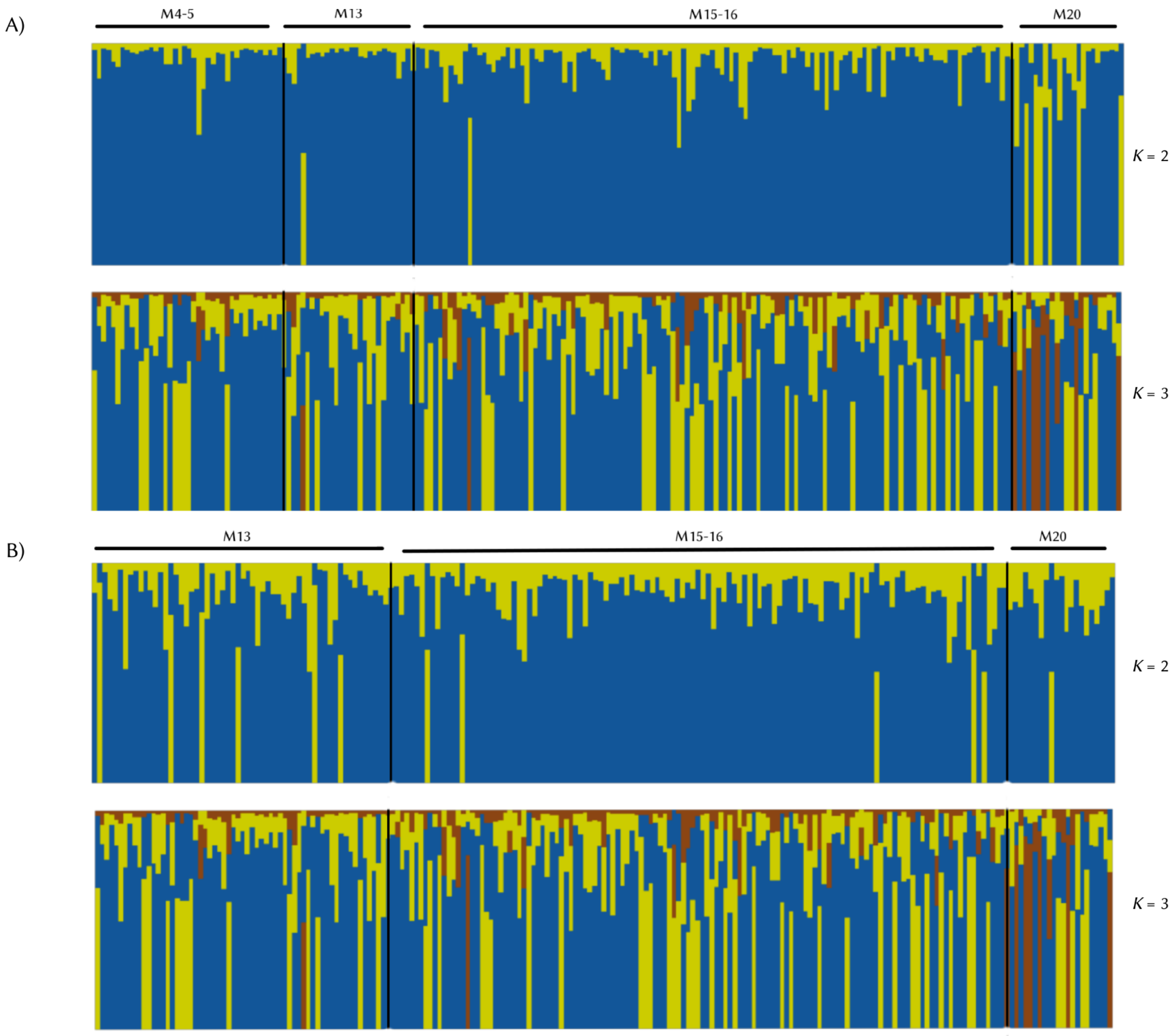

Figure S2. Genetic structure of *M. ganzhorni* in forest fragments sampled in the littoral rain forest of Mandena. STRUCTURE diagrams correspond to the full dataset with uneven sample sizes among fragments illustrating membership for each individual for *K*=2 and K=3. A) 1998-2003 dataset (M4-5, M13, M15-16 and M20) and B) 2012-2016 dataset (M13, M15-16 and M20) the establishment of corridors in the littoral rain forest of Mandena.

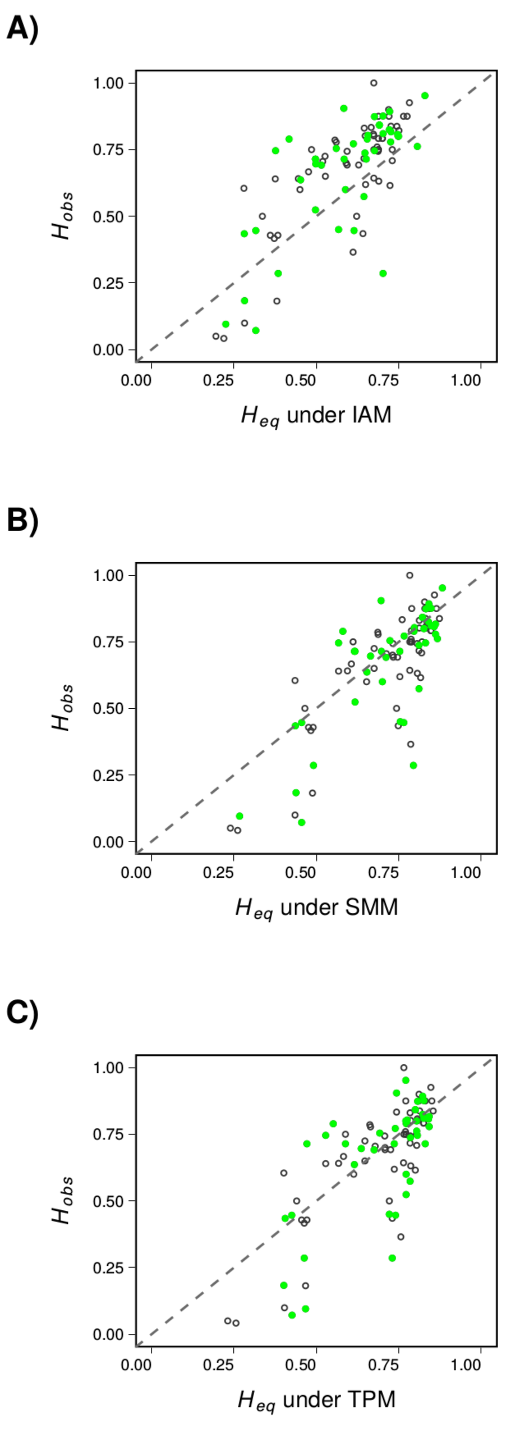

Figure S3. Gene diversity (H_{obs}) versus equilibrium gene diversity (H_{eq}) of *M. ganzhorni* predicted by the number of alleles observed before (open circles) and after (green circles) the establishment of corridors between forests fragments (M13, M15-16 and M20) of Mandena. Each point represent a microsatellite locus (N=14).

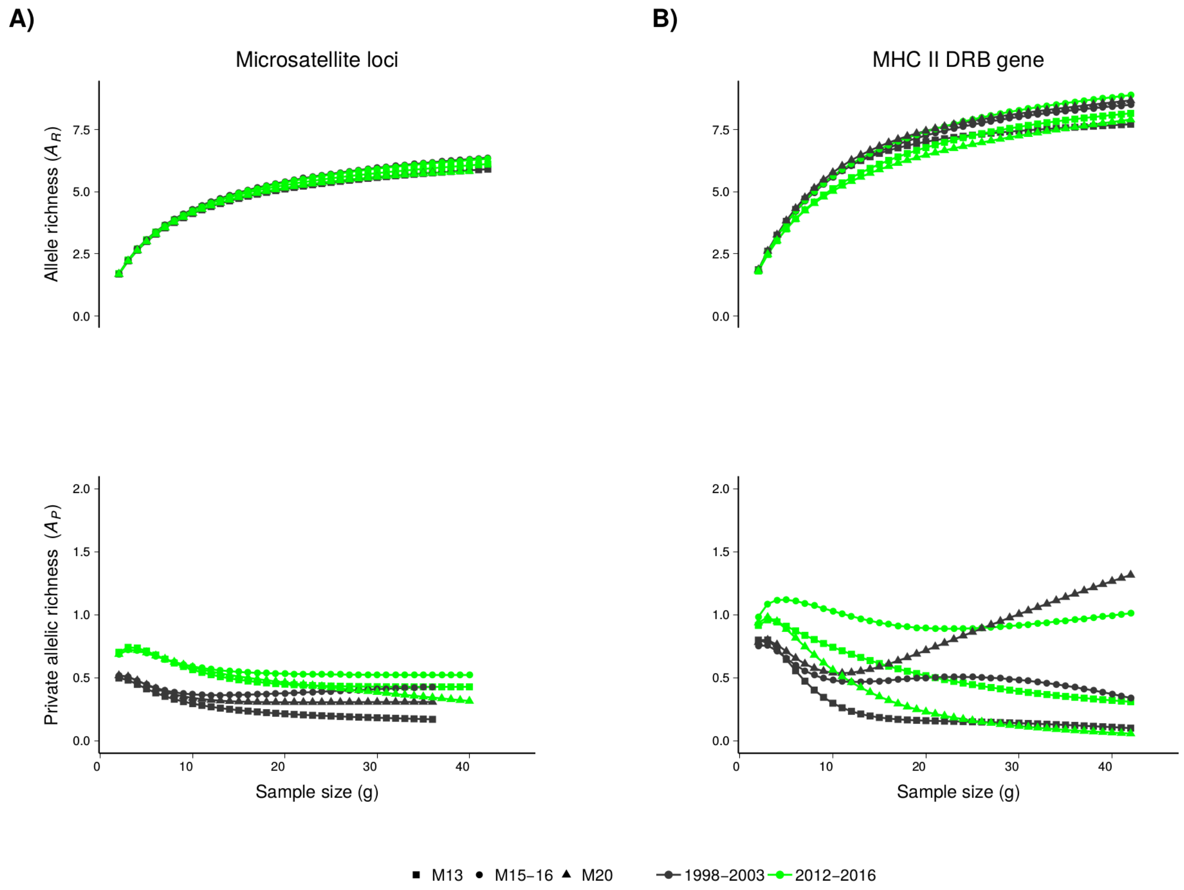

Supplementary Figure S4. Inference of mean number of distinct alleles (AR) and mean number of private alleles (AP) per locus using the rarefaction method in ADZE software (Szpiech et al. 2008). Forest fragment M4-5 was cleared by 2012 and therefore was excluded from the analyses.

.

5. Supplemental references

Babik, W. (2010). Methods for MHC genotyping in non-model vertebrates. *Molecular Ecology Resources, 10*(2), 237–251. doi:10.1111/j.1755-0998.2009.02788.x

Brakenhoff, R. H., Schoenmakers, J. G., & Lubsen, N. H. (1991). Chimeric cDNA clones: a novel PCR artifact. *Nucleic Acids Research, 19*(8), 1949.

Burri, R., Promerová, M., Goebel, J., & Fumagalli, L. (2014). PCR-based isolation of multigene families: lessons from the avian MHC class IIB. *Molecular Ecology Resources*, *14*(4), 778–788. doi:10.1111/1755-0998.12234

Edgar, R. C., Haas, B. J., Clemente, J. C., Quince, C., & Knight, R. (2011). UCHIME improves sensitivity and speed of chimera detection. *Bioinformatics*, *27*(16), 2194–2200. doi:10.1093/bioinformatics/btr381

Eren, A. M., Morrison, H. G., Lescault, P. J., Reveillaud, J., Vineis, J. H., & Sogin, M. L. (2015). Minimum entropy decomposition: Unsupervised oligotyping for sensitive partitioning of high-throughput marker gene sequences. *The ISME Journal*, *9*(4), 968–979. doi:10.1038/ismej.2014.195

Evanno, G., Regnaut, S., & Goudet, J. (2005). Detecting the number of clusters of individuals using the software structure: a simulation study. *Molecular Ecology*, *14*(8), 2611–2620. doi:10.1111/j.1365-294X.2005.02553.x

Galan, M., Guivier, E., Caraux, G., Charbonnel, N., & Cosson, J.-F. (2010). A 454 multiplex sequencing method for rapid and reliable genotyping of highly polymorphic genes in large-scale studies. *Bmc Genomics*, *11*(1), 1.

Glavač, D., & Dean, M. (1993). Optimization of the single-strand conformation polymorphism (SSCP) technique for detection of point mutations. *Human Mutation*, *2*(5), 404–414. doi:10.1002/humu.1380020513

Glenn, T. C. (2011). Field guide to next-generation DNA sequencers. *Molecular Ecology Resources*, *11*(5), 759–769. doi:10.1111/j.1755-0998.2011.03024.x

Hayashi, K., & Yandell, D. W. (1993). How sensitive is PCR-SSCP? *Human Mutation*, *2*(5), 338–346. doi:10.1002/humu.1380020503

Huchard, E., Albrecht, C., Schliehe-Diecks, S., Baniel, A., Roos, C., Peter, P. M. K., & Brameier,

M. (2012). Large-scale MHC class II genotyping of a wild lemur population by next generation sequencing. *Immunogenetics*, *64*(12), 895–913. doi:10.1007/s00251-012-0649-6

Lenz, T. L., & Becker, S. (2008). Simple approach to reduce PCR artefact formation leads to reliable genotyping of MHC and other highly polymorphic loci — Implications for evolutionary analysis. *Gene*, *427*(1–2), 117–123. doi:10.1016/j.gene.2008.09.013

Lighten, J., van Oosterhout, C., & Bentzen, P. (2014). Critical review of NGS analyses for de novo genotyping multigene families. *Molecular Ecology*, *23*(16), 3957–3972. doi:10.1111/mec.12843

Magoč, T., & Salzberg, S. L. (2011). FLASH: fast length adjustment of short reads to improve genome assemblies. *Bioinformatics*, *27*(21), 2957–2963. doi:10.1093/bioinformatics/btr507

Meyerhans, A., Vartanian, J.-P., & Wain-Hobson, S. (1990). DNA recombination during PCR. *Nucleic Acids Research*, *18*(7), 1687–1691. doi:10.1093/nar/18.7.1687

Ortí, G., Hare, M. P., & Avise, J. C. (1997). Detection and isolation of nuclear haplotypes by PCR-SSCP. *Molecular Ecology*, *6*(6), 575–580.

Pritchard, J. K., Stephens, M., & Donnelly, P. (2000). Inference of population structure using multilocus genotype data. *Genetics*, *155*(2), 945–959.

Puechmaille, S. J. (2016). The program structure does not reliably recover the correct population structure when sampling is uneven: subsampling and new estimators alleviate the problem. *Molecular Ecology Resources*, *16*(3), 608–627. doi:10.1111/1755-0998.12512

Qiu, X., Wu, L., Huang, H., McDonel, P. E., Palumbo, A. V., Tiedje, J. M., & Zhou, J. (2001). Evaluation of PCR-Generated Chimeras, Mutations, and Heteroduplexes with 16S rRNA Gene-Based Cloning. *Applied and Environmental Microbiology*, *67*(2), 880–887. doi:10.1128/AEM.67.2.880-887.2001

Radwan, J., Zagalska-Neubauer, M., Cichoń, M., Sendecka, J., Kulma, K., Gustafsson, L., & Babik, W. (2012). MHC diversity, malaria and lifetime reproductive success in collared flycatchers. *Molecular Ecology*, *21*(10), 2469–2479. doi:10.1111/j.1365-294X.2012.05547.x

Santos, P. S. C., Courtiol, A., Heidel, A. J., Höner, O. P., Heckmann, I., Nagy, M., … Sommer, S. (2016). MHC-dependent mate choice is linked to a trace-amine-associated receptor gene in a mammal. *Scientific Reports*, *6*, 38490. doi:10.1038/srep38490

Schad, J., Ganzhorn, J. U., & Sommer, S. (2005). Parasite burden and constitution of major

histocompatibility complex in the Malagasy mouse lemur, Microcebus murinus. *Evolution*, *59*(2), 439–450.

Schad, J., Sommer, S., & Ganzhorn, J. U. (2004). MHC variability of a small lemur in the littoral forest fragments of southeastern Madagascar. *Conservation Genetics*, *5*(3), 299–309.

Schuelke, M. (2000). An economic method for the fluorescent labeling of PCR fragments. *Nature Biotechnology*, *18*(2), 233–234.

Sebastian, A., Herdegen, M., Migalska, M., & Radwan, J. (2016). AMPLISAS : a web server for multilocus genotyping using next-generation amplicon sequencing data. *Molecular Ecology Resources*, *16*(2), 498–510. doi:10.1111/1755-0998.12453

Smyth, R. P., Schlub, T. E., Grimm, A., Venturi, V., Chopra, A., Mallal, S., … Mak, J. (2010). Reducing chimera formation during PCR amplification to ensure accurate genotyping. *Gene*, *469*(1–2), 45–51. doi:10.1016/j.gene.2010.08.009

Sommer, S., Courtiol, A., & Mazzoni, C. J. (2013). MHC genotyping of non-model organisms using next-generation sequencing: a new methodology to deal with artefacts and allelic dropout. *BMC Genomics*, *14*(1), 1.

Sunnucks, P., Wilson, A. C. C., Beheregaray, L. B., Zenger, K., French, J., & Taylor, A. C. (2000). SSCP is not so difficult: the application and utility of single-stranded conformation polymorphism in evolutionary biology and molecular ecology. *Molecular Ecology*, *9*(11), 1699–1710. doi:10.1046/j.1365-294x.2000.01084.x

van Oosterhout, C., Hutchinson, W. F., Wills, D. P. M., & Shipley, P. (2004). micro-checker: software for identifying and correcting genotyping errors in microsatellite data. *Molecular Ecology Notes*, *4*(3), 535–538. doi:10.1111/j.1471-8286.2004.00684.x
